# Identification of Drought Tolerance Markers in a Diverse Population of Rice Cultivars by Expression and Metabolite Profiling

**DOI:** 10.1371/journal.pone.0063637

**Published:** 2013-05-22

**Authors:** Thomas Degenkolbe, Phuc T. Do, Joachim Kopka, Ellen Zuther, Dirk K. Hincha, Karin I. Köhl

**Affiliations:** 1 Max Planck Institute of Molecular Plant Physiology, Potsdam, Brandenburg, Germany; University of Delhi South Campus, India

## Abstract

Rice provides about half of the calories consumed in Asian countries, but its productivity is often reduced by drought, especially when grown under rain-fed conditions. Cultivars with increased drought tolerance have been bred over centuries. Slow selection for drought tolerance on the basis of phenotypic traits may be accelerated by using molecular markers identified through expression and metabolic profiling. Previously, we identified 46 candidate genes with significant genotype × environment interaction in an expression profiling study on four cultivars with contrasting drought tolerance. These potential markers and in addition GC-MS quantified metabolites were tested in 21 cultivars from both *indica* and *japonica* background that varied in drought tolerance. Leaf blades were sampled from this population of cultivars grown under control or long-term drought condition and subjected to expression analysis by qRT-PCR and metabolite profiling. Under drought stress, metabolite levels correlated mainly negatively with performance parameters, but eight metabolites correlated positively. For 28 genes, a significant correlation between expression level and performance under drought was confirmed. Negative correlations were predominant. Among those with significant positive correlation was the gene coding for a cytosolic fructose-1,6-bisphosphatase. This enzyme catalyzes a highly regulated step in C-metabolism. The metabolic and transcript marker candidates for drought tolerance were identified in a highly diverse population of cultivars. Thus, these markers may be used to select for tolerance in a wide range of rice germplasms.

## Introduction

Rice (*Oryza sativa* L.) is one of the world’s most important staple foods with 720 million tons harvested in 2011 (www.fao.org 24.07.2012). In Asia, its main cultivation area, rice provides 35–60% of the calories consumed [1]. Rice was domesticated at least twice independently, which resulted in the two subspecies *indica* and *japonica* [2]. Centuries of breeding furthermore yielded a wide range of cultivars adapted to different watering regimes from irrigated, deep-water cultures to rain-fed lowland and upland cultivars [3]. About 50% of the rice acreage is rain-fed and not irrigated [4]. In these areas, drought is the major environmental factor that reduces productivity by 13–35% [5], [6]. Drought stress causes yield loss not only in rice, but in many other crops like potato, wheat and maize. The situation will aggravate in future as agriculture competes with others consumers for limited water supplies. Thus, more food will have to be produced with less water to provide for the increasing world population [7].

Therefore, strategies to identify drought-tolerant germplasms are of major interest. Traditionally, breeding of drought-tolerant cultivars relied on selection based on phenotypic and physiological traits observed under drought stress [8], [9], namely leaf rolling [10]–[12], cell membrane stability [13], carbon isotope discrimination, gas exchange and chlorophyll fluorescence measurements [14]–[19], stomatal conductance and water use efficiency [19], [20], root traits [21] and yield [22], [23]. However, this selection process is labour-intensive and slow as it requires cultivation of breeding populations under drought conditions [24]. The phenotypic evaluation can, however, be replaced by the use of molecular markers such as DNA polymorphisms or chemical tags [25] associated with the trait. Marker-assisted selection (MAS) is cheaper and more convenient than phenotype-based selection and it presently is the only option to combine traits by gene pyramiding [25]. DNA based markers can be derived from quantitative trait loci (QTL) and allow selection already in the seedling stage. QTLs for drought tolerance traits have been identified in the last decade in rice [12], [13], [23], [26]–[30], wheat [31], [32], maize [33], [34] and other crops. New breeding markers based on transcript or metabolite abundance can be derived from multi-parallel methods like expression and metabolic profiling [3]. For complex traits like drought tolerance, studies have shown that markers will indicate traits contributing to drought tolerance rather than overall tolerance [25]. Thus, ideally, the concentration of a marker transcript or metabolite will correlate with one or several traits contributing to drought tolerance in a wide range of cultivars.

For our marker search, we used metabolite and expression profiling. Metabolite were measured on the Golm Metabolomics platform [35]. In a previous microarray study, we have identified genes that were differentially expressed in four rice genotypes of contrasting drought tolerance [36] and thus could be marker candidates for drought tolerance. As the ideal marker should correlate positively with drought tolerance in a wide range of genetic backgrounds, we tested the potential markers in an association type study. We choose *indica* and *japonica* cultivars that originated from a variety of Vietnamese agro-ecosystems and had been selected into a breeding program for drought and salt stress. Some well characterised cultivars from the International Rice Research Institute (IRRI, Manila, Philippines) were included additionally. All cultivars have been characterised for several traits related to drought tolerance under control and long-term drought stress in a parallel study (Do et al. PLOS ONE 10.1371/journal.pone.0060325). In the present study, we checked, which RNA and metabolite levels allowed prediction of drought tolerance related traits. These transcripts and metabolites may be drought tolerance markers in rice.

## Results

### Genotyping of Cultivars

In our study, 17 of the 21 cultivars (Table 1) originated from a Vietnamese breeding program for drought stress resistance. As information on the pedigree of these cultivars was limited, six subspecies-specific sequence tagged sites (STS) markers located on four chromosomes [37] were chosen to determine to which subspecies (*japonica* or *indica*) the cultivars belong (Table 1). Cultivars with known pedigree (*japonica* cultivars 50, 51 and 54, *indica* cultivars 52, 55 and 62) were included as references. Based on the results, three of the Vietnamese cultivars were classified as *japonica* and eight as *indica*. Four cultivars (3, 13, 15 and 17) were mainly *japonica* with some *indica* introgression. Two cultivars (14 and 18) were classified as mainly *indica* with some *japonica* introgression. Thus, the studied genotypes represent both the *indica* and *japonica* gene pools.

**Table 1:** Rice cultivars in study population belong to indica or japonica subspecies.

		Marker Name					
Cultivar	ID	S01022	S03020	S03136	S04128	S07011	S07103
CR203	1	*ind.*	*ind.*	*ind.*	*ind.*	*ind.*	*ind.*
DR2	2	*ind.*	*ind.*	*ind.*	*ind.*	*ind.*	*ind.*
Loc	3	**jap.**	**jap.**	**jap.**	*ind.*	**jap.**	**jap.**
C70	4	*ind.*	*ind.*	*ind.*	*ind.*	*ind.*	*ind.*
C71	5	*ind.*	*ind.*	*ind.*	*ind.*	*ind.*	*ind.*
K.lua nuong	13	**jap.**	**jap.**	**jap.**	*ind.*	**jap.**	**jap.**
Cuom	14	**jap.**	*ind.*	*ind.*	*ind.*	*ind.*	**jap.**
Khau cham	15	**jap.**	**jap.**	**jap.**	*ind.*	**jap.**	**jap.**
Khau hom	16	**jap.**	**jap.**	**jap.**	**jap.**	**jap.**	**jap.**
Khau non	17	**jap.**	**jap.**	**jap.**	*ind.*	**jap.**	**jap.**
Tra linh	18	*ind.*	*ind.*	*ind.*	*ind.*	*ind.*	**jap.**
Nep men	19	*ind.*	*ind.*	*ind.*	*ind.*	*ind.*	*ind.*
Loc dau	20	*ind.*	*ind.*	*ind.*	*ind.*	*ind.*	*ind.*
Lua man	22	*ind.*	*ind.*	*ind.*	*ind.*	*ind.*	*ind.*
LC-93-1	29	**jap.**	**jap.**	**jap.**	**jap.**	**jap.**	**jap.**
LC-93-2	30	*ind.*	*ind.*	*ind.*	*ind.*	*ind.*	*ind.*
LC-93-4	31	**jap.**	**jap.**	**jap.**	**jap.**	**jap.**	**jap.**
**Nipponbare**	50	**jap.**	**jap.**	**jap.**	**jap.**	**jap.**	**jap.**
**Taipei309**	51	**jap.**	**jap.**	**jap.**	**jap.**	**jap.**	**jap.**
IR57311-95-2-3	52	*ind.*	*ind.*	*ind.*	*ind.*	*ind.*	*ind.*
Zonghua	53	**jap.**	**jap.**	**jap.**	**jap.**	**jap.**	**jap.**
**CT 9993-5-10-1**	54	**jap.**	**jap.**	**jap.**	**jap.**	**jap.**	**jap.**
**IR 62266-42-6-2**	55	*ind.*	*ind.*	*ind.*	*ind.*	*ind.*	*ind.*
**IR 64**	62	*ind.*	*ind.*	*ind.*	*ind.*	*ind.*	*ind.*

Genotyping of rice cultivars based on the amplification of six subspecies-specific sequence tagged sites (STS) markers. *ind.* – *indica*, **jap.** – *japonica*. Bold print of cultivar name: reference cultivars with known genotype.

### Identification of Potential Gene Expression-based Markers

**Figure 1:**
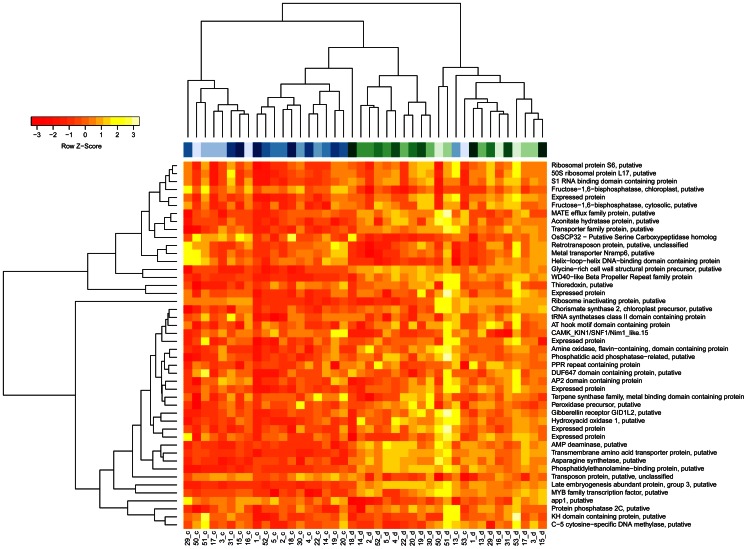
RNA expression response to drought differs between rice cultivars. Hierarchical clustering and heatmap of expression levels in leaves of rice plants grown under control conditions (c) or drought stress (d). The code below the heatmap indicates the line id (see Table 1) and the condition (blue: control = c, green: drought stress = d).

Potential marker genes had been identified in an expression profiling study on four rice genotypes of contrasting drought tolerance [36]. Genes had been selected as marker candidates, when their expression response to drought stress differed between tolerant and sensitive cultivars. This response pattern was identified in an analysis of variance by a significant interaction effect of the factors condition (drought, control) and tolerance group (sensitive cultivars, tolerant cultivars) on gene expression [36]. To reduce the list of candidates to those with agricultural relevance, we have compared the position of the candidate gene to the positions of published QTL for drought tolerance in rice [36]. From 108 candidate genes with significant interaction and location in a published QTL, we have chosen the 46 genes with the lowest p-values for further analysis in the present study. Expression levels of these genes were measured by qRT-PCR in leaf blades of 21 rice cultivars grown under control and drought stress condition identical to those in the previous microarray study (see Table S1 for all qRT-PCR results). Hierarchical clustering of expression patterns in biological samples (Figure 1) separated samples from well-watered and drought treated plants neatly. The few exceptions were from extreme cultivars. The highly sensitive cultivar 53 showed a drought stress expression pattern already under control conditions. In contrast, under drought stress the expression pattern of the highly tolerant cultivar 18 resembled the expression pattern found in other cultivars under well-watered conditions. Most marker candidates were more highly expressed under drought stress than under control conditions.

**Figure 2:**
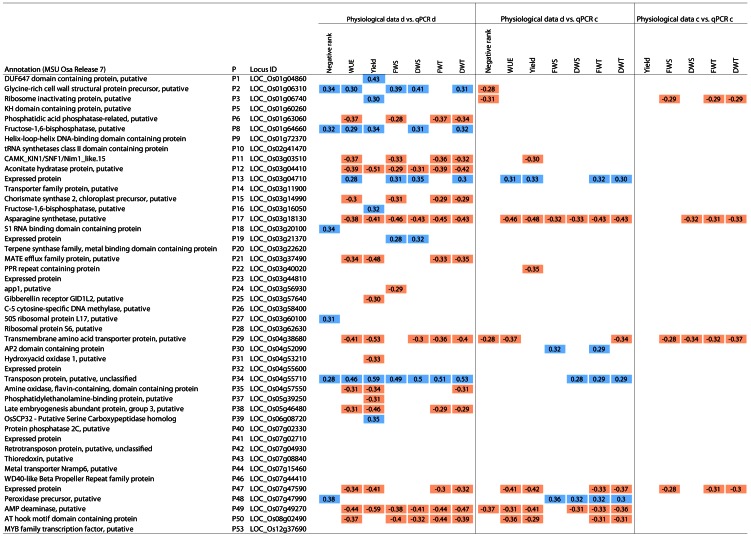
Correlation of physiological data with candidate gene expression. Annotation, primer (P), locus identifier of and correlation coefficients for candidate genes with significant (p<0.05) positive (blue) or negative (red) correlation of log-transformed expressions levels with physiological data under drought (d) or control (c) conditions. Data of 21 different cultivars with 2 to 3 replicates per cultivar and condition, 51 data pairs in total. Negative rank - mean scoring rank multiplied with -1; WUE - water use efficiency; yield - chlorophyll-*a* fluorescence yield; FWS - fresh weight shoot; DWS - dry weight shoot; FWT - total fresh weight; DWT - total dry weight. Sorted by LocusID.

To identify markers, correlations between relative expression levels of the candidate genes and physiological parameters contributing to performance under drought were determined. These traits were assessed on vegetative plants after 18 days of growth under control or drought stress conditions (see the accompanying paper Do et al. PLOS ONE 10.1371/journal.pone.0060325). Under drought stress, the parameters drought score (representing the stay-green trait) and mean water use efficiency (WUE) were determined. Additionally, correlations to shoot fresh and dry weight, total fresh and dry weight and photosynthetic yield (measured as photosystem II quantum use efficiency by chlorophyll fluorescence spectroscopy) under control and drought stress were analysed (Figure 2). Parameters were mathematically transformed to ensure that high parameter values indicate good performance (see Methods, ‘Correlation analyses). Ideally, correlation to all performance parameters should show the same direction if the candidate gene expression is a good predictor for several tolerance traits. Under drought stress, expression levels of 28 of the 46 candidate genes correlated significantly (p<0.05) with physiological performance parameters under drought stress (Figure 2).

**Figure 3:**
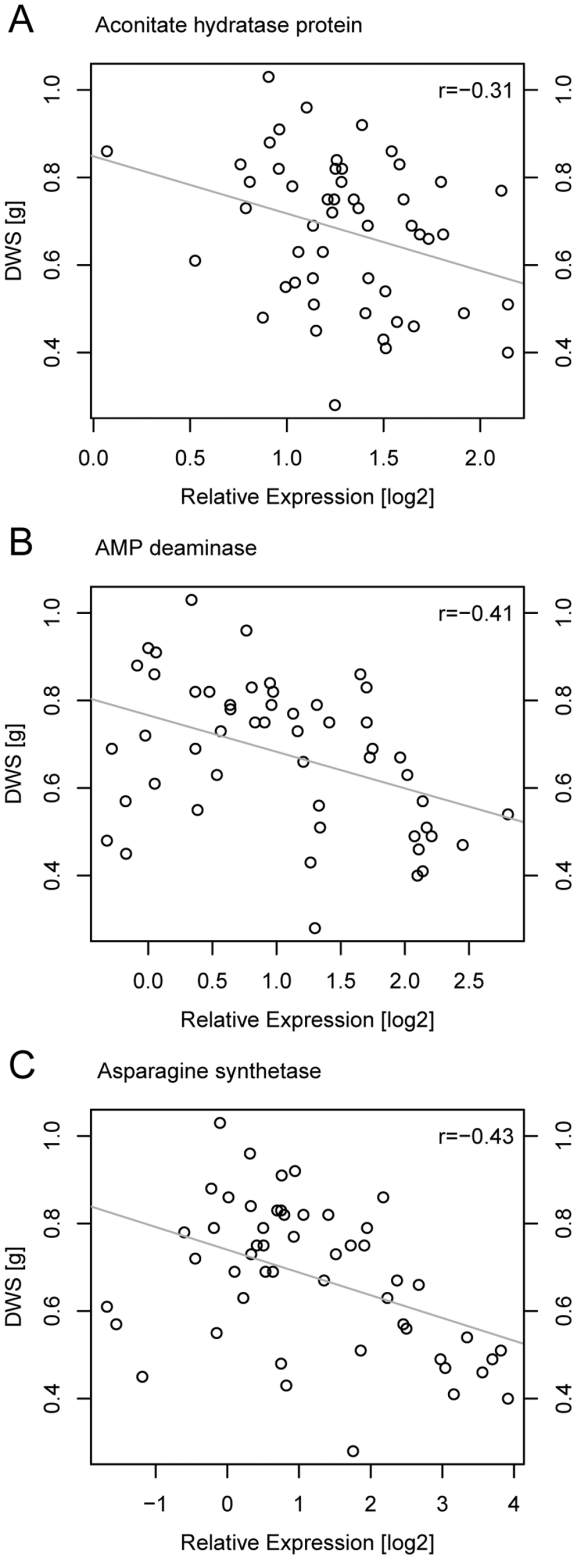
Expression of genes for aconitate hydratase (A), an AMP deaminase (B) and asparagine synthetase (C) correlated negatively with shoot dry weight. Relative expression levels of genes and average shoot dry weight measured in rice cultivars in three independent experiments. The regression coefficient r for the linear regression of shoot dry weight against expression level is shown in the upper right corner.

For 11 genes, expression under drought stress correlated negatively with several phenotypic parameters, indicating high expression levels in cultivars with poor performance. The expression of genes coding for an aconitate hydratase, an AMP deaminase and an asparagine synthetase correlated negatively with most performance parameters under drought stress (s. Figure 3). Also under control conditions, expression of asparagine synthase and AMP deaminase genes correlated negatively with performance parameters under drought. Both genes were thus more strongly expressed in drought-sensitive cultivars than in tolerant cultivars under water-sufficient and water-deficient conditions, suggesting a constitutive increase in the expression of these genes in drought-sensitive compared to drought-tolerant cultivars. Expression of the asparagine synthetase gene and genes encoding a transmembrane amino acid transporter protein and an expressed protein at LOC_Os07g47590 also correlated negatively with performance parameters under control conditions. High expression levels of these genes thus indicated slow growth rather than poor performance especially under drought. For the other genes, expression under control conditions did not correlate with growth or photosynthesis under control conditions. This suggests that the difference in gene expression between cultivars was not linked to general differences in growth rates. High expression levels of these genes indicated drought sensitivity.

**Figure 4:**
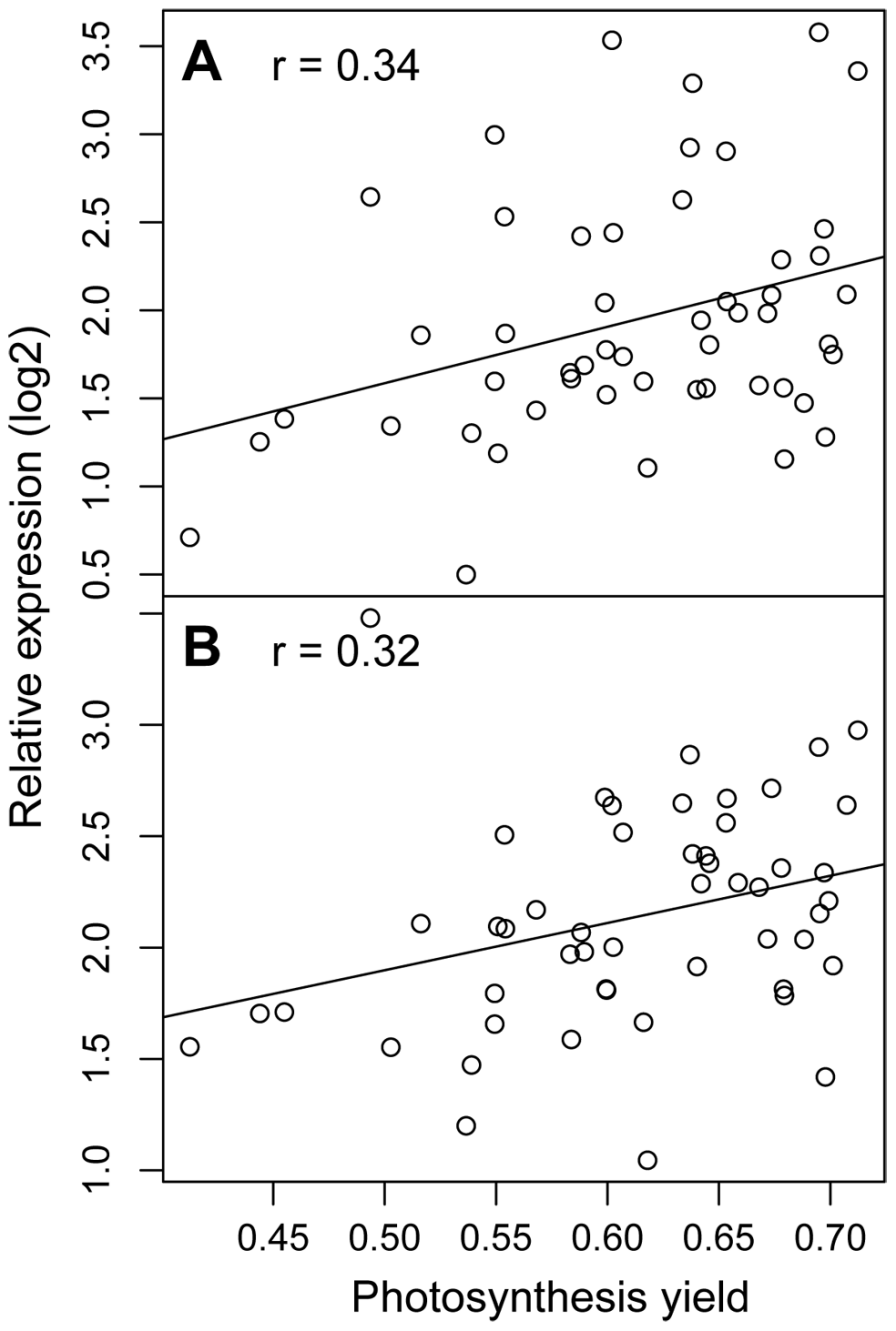
Expression of the fructose-1,6-bisphosphatase gene correlated with photosynthetic quantum yield. Correlation of relative expression levels of genes coding for a cytosolic (A) and a plastidial (B) fructose-1,6-bisphosphatase with photosynthetic quantum yield of leaves measured after 18 days of growth under drought stress.

Significant positive correlations between expression levels under drought and drought score were found for six candidate genes (Figure 2). The expression of these genes could serve as tolerance markers. For three of these genes, expression levels were also positively correlated with shoot and total dry weight under drought conditions (Figure 2 and Figure S1). These genes encode a cytosolic fructose-1,6-bisphosphatase, a glycine-rich cell wall structural protein, and a transposon protein. For these genes, high expression levels under drought stress indicated high drought tolerance. Their expression levels under control conditions showed only a few significant correlations to performance parameters under drought stress. Thus, in contrast to the constitutive sensitivity markers (e.g. asparagine synthase gene and an AMP deaminase encoding gene, see above), expression of most tolerance marker genes seemed to be drought-induced, i.e. their expression levels could only be used as markers under drought stress. Both, the cytosolic fructose-1,6-bisphosphatase and the plastidial precursor of fructose-1,6-bisphosphatase correlated significantly with the photosynthetic yield (Figure 4).

### Identification of Potential Metabolic Markers

**Figure 5:**
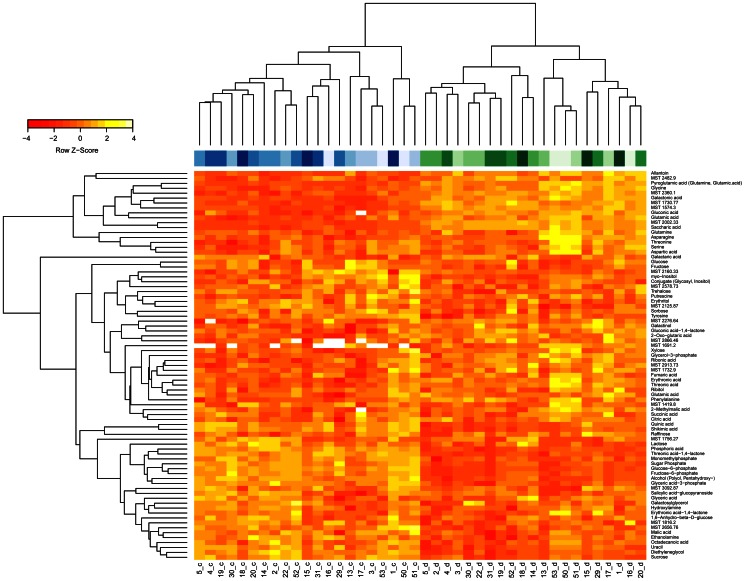
Metabolite response to drought differs between rice cultivars. Hierarchical clustering and heatmap of metabolite levels in leaves of rice plants grown under control conditions (c) or drought stress (d). Metabolite levels were normalised within an experiment by Z-transformation as indicated in Material and Methods. The code below the heatmap indicates the line id (see Table 1) and the condition (blue: control, green: drought stress).

Metabolite levels were determined in leaves of control and drought-stressed plants from 21 cultivars (see Table S2). Hierarchical clustering for both metabolites and samples (cultivars × condition) are shown in Figure 5. The clustering of samples showed a complete separation of the metabolite pattern between samples from control and drought-treated material. The drought treatment was thus the main source of variance in the data, which indicates a complete change of metabolism under stress conditions in all cultivars. The changes induced by the treatment are predominantly larger than the differences between cultivars within a treatment. The upper cluster in the metabolite hierarchy shown in Figure 5 contains metabolites that increased under drought stress; it contains glutamine, glutamic acid and derivatives. In contrast, metabolites grouping with sugar phosphates (lowest cluster in Figure 5) decreased under drought stress.

**Figure 6:**
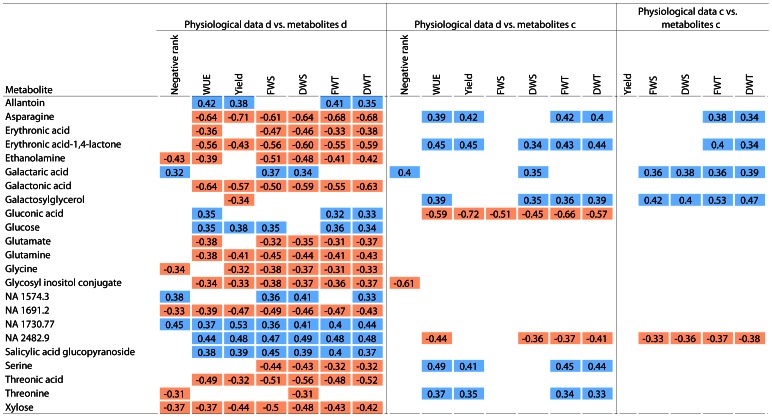
Correlation of physiological data with metabolite levels. Correlation coefficients for selected metabolites with significant (p<0.05) positive (blue) or negative (red) correlation between log-transformed metabolite levels with physiological data under drought (d) or control (c) conditions. Data of 21 different cultivars grown in two experiments. Mean values of three to five replicates per cultivar and condition and experiment were correlated. Negative rank - mean scoring rank multiplied with -1; WUE – water use efficiency (g DW/g H_2_O per day); yield - chlorophyll-*a* fluorescence yield; FWS - fresh weight shoot [g]; DWS - dry weight shoot [g]; FWT - total fresh weight [g]; DWT - total dry weight [g].

**Figure 7:**
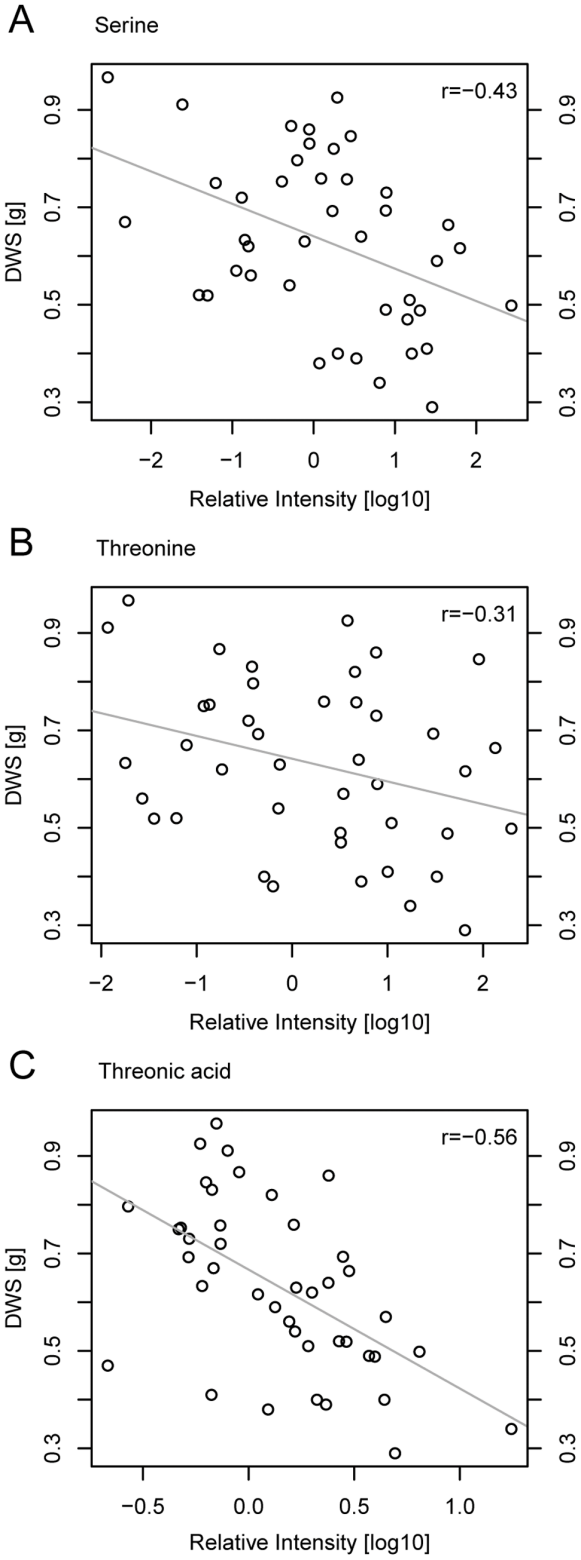
Levels of serine (A) threonine (B) and threonic acid (C) correlated negatively with shoot dry weight of rice plants under drought stress. Average metabolite levels and average shoot dry weight of 20 rice cultivars from two independent experiments.

**Figure 8:**
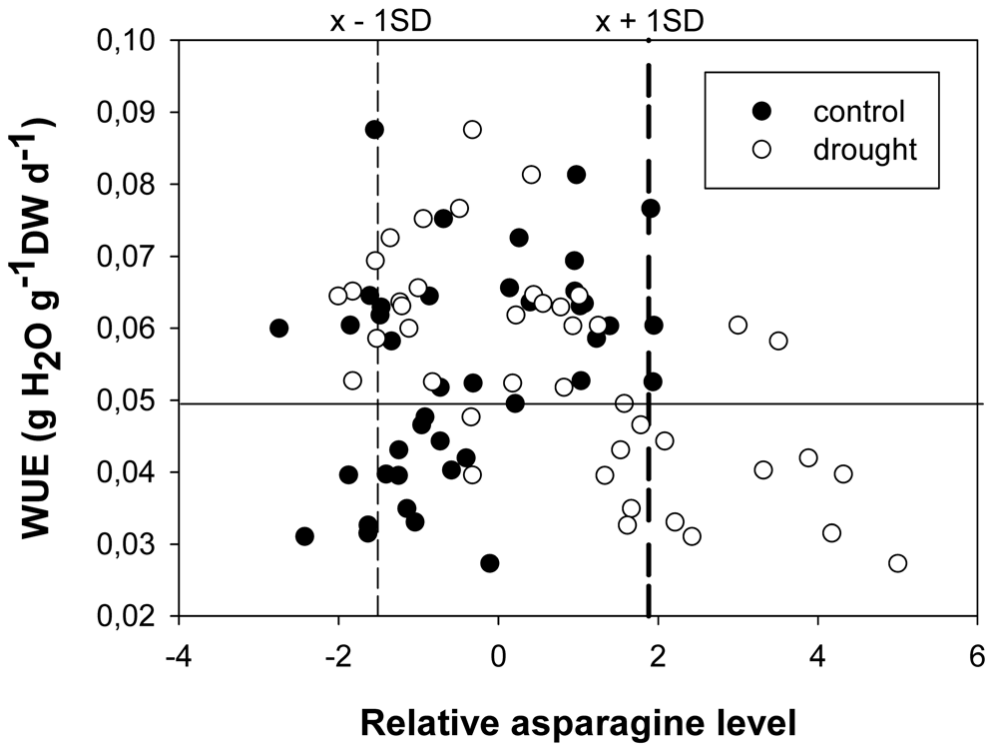
High asparagine levels are predominantly found in cultivars with low water use efficiency. Water use efficiency (g water per g final dry weight per day) of rice cultivars grown under control or drought conditions plotted against the relative asparagine level (Z score of log2 transformed values) in their leaves. The vertical reference lines indicate the average asparagine level of all samples minus (left) or plus (right) one standard deviation.

Most of the significant correlations between metabolite levels and performance parameters were negative under drought stress (Figure 6). Negative correlations were found for the concentration of the amino acids asparagine, glutamate, glutamine, glycine, serine and threonine, and for the organic acids erythronic, galactonic and threonic acid. Higher concentrations of these metabolites were connected with lower fresh and dry weight, lower photosynthetic quantum yield and lower water use efficiency. In sensitive cultivars, levels were 10 to 100 fold higher than in tolerant cultivars (see Figure 7 and Figure S2, notice log-10 scale for metabolite levels). Under control conditions, levels of asparagine, erythronic acid-1,4-lactone, serine and threonine correlated positively with performance under drought. Asparagine, threonine and serine levels were significantly higher (p<0.05) under drought than under control conditions. Those cultivars that accumulated asparagine more than most other cultivars (levels above mean plus one standard deviation) predominantly showed a below than average water use efficiency (WUE) (Figure 8). Thus, high asparagine levels indicated low WUE.

**Figure 9:**
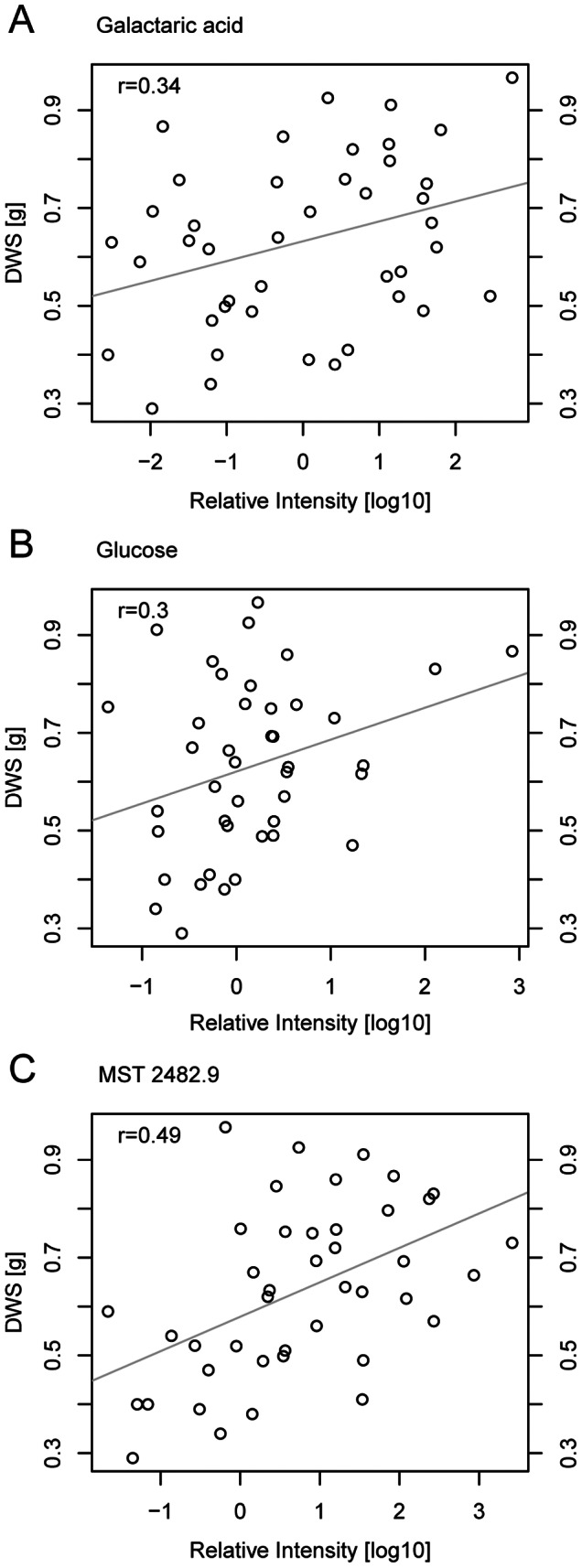
Levels of galactaric acid (A), glucose (B) and MST 2482.9 (C) correlate positively with shoot dry weight of rice plants under drought stress. Average metabolite levels and average shoot dry weight of 20 rice cultivars from two independent experiments.

Positive correlations between metabolite levels and drought tolerance traits were identified for allantoin, galactaric acid, gluconic acid, glucose, a salicylic acid glucopyranoside and three unknown analytes with a retention time index of 1574.3, 1730.77 and 2482.9 (Figure 6, Figure 9 and Figure S2). Concentrations of these metabolites were 10 to 1000fold higher in tolerant plants than in sensitive plants. Under drought stress, levels of these metabolites were high in tolerant cultivars. However, for most of these metabolites no correlations between levels under control conditions and performance under drought were found. In contrast, galactaric acid concentrations under control conditions correlated positively with the performance under drought. As levels under control conditions correlated positively with growth under control conditions too, galactaric acid levels seemed to relate to growth rate rather than to drought tolerance. Glucose and gluconic acid, for which positive correlations between concentration and performance were restricted to drought stress conditions, were thus better marker candidates.

### Principal Component Analysis

**Figure 10:**
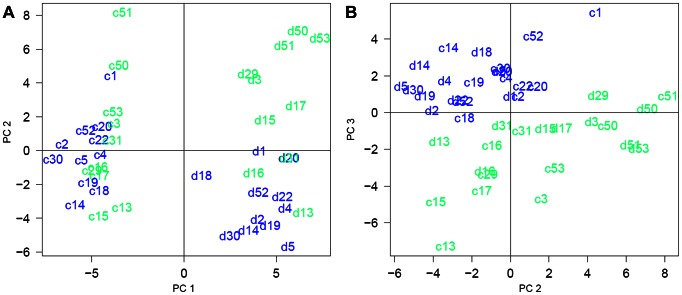
Principal component analysis (PCA) separated samples by treatment and genetic origin of rice cultivars. PCA plots on normalised metabolite levels with PC1 and PC2 (A) separated samples of plants grown under control (c) and drought conditions (d). PC2 and PC3 (B) separated samples of *indica* (blue) and *japonica* cultivars (green). Numbers for cultivars see Table 1.

Improved prediction of drought tolerance might be gained from derived variables e.g. from linear combinations of gene expression or metabolite concentration values. To that end, we checked whether the variation in drought tolerance can be resolved by a combination of principle components on metabolite concentrations. In a dataset from leaves of control and drought-stressed plants grown in two experiments, component 1 (PC1) separated control and drought-stressed plants (Figure 10A). This component explained 31% of the variance. PC1 was a linear combination of many metabolites without obvious overrepresentation of metabolites from a single pathway (Table S3). A combination of PC2 and PC3, explaining 16% and 9% of the variance, respectively, separated *japonica* and *indica* cultivars (Figure 10B). In PC2, erythronic acid-1,4-lactone and three amino acids (aspartate, serine and threonine) had loadings higher than 0.2. However, tolerance differences between the cultivars could not be resolved in one of the PC-plots. Likewise, multiple regression approaches (data not shown) yielded poorly reproducible results that were highly dependent on the regression method and the quality criterion. Thus, no combined markers based on multiple metabolite levels could be gained from the data.

## Discussion

Drought tolerance in crops is an increasingly relevant trait, as water availability is the limiting factor for plant production especially in those parts of the world, in Asia and in Africa, where malnutrition is a major issue. However, drought tolerance is a quantitative agricultural trait that is very difficult and labour-intensive to determine. In the past, drought tolerance has been assessed in field trials to measure either final yield or physiological parameters that are predictive for yield under stress. Yield itself is the most relevant parameter, but its heritability is regrettably low. Additionally, drought tolerance depends very much on the target environment. Thus, marker search concentrates on features that predict traits contributing to drought tolerance in a defined environment [8], [25]. One of these traits is the stay-green trait that estimates the degree of leaf chlorosis and necrosis [9] under stress. The ability to maintain a high biomass under drought stress at the juvenile stage enhances plant survival after transplanting as well as rapid recovery after drought. Both features increase yield.

We used the stay-green trait measured as drought score categories as the main trait for the quantification of drought tolerance in our test population and tested the predictability of this from transcript or metabolite data. Additionally, we checked whether we can predict further traits relevant for drought tolerance, such as chlorophyll fluorescence yield [38], water use efficiency, or total and shoot biomass. In QTL studies, the association between genes or genomic markers and various proxy-parameters for drought tolerance is not consistent [32], [39]. In our study, some metabolites like asparagine concentrations and some transcript levels e.g. of asparagine synthetase correlated closely to several traits, whereas others marker candidates correlated specifically to e.g. water use efficiency or chlorophyll fluorescence.

To speed up breeding by marker assisted selection (MAS), markers should allow tolerance prediction from features that can be measured on young plants, ideally without the need of prior stress treatment [19], [22]. In contrast to genomic markers, metabolite and transcript levels vary with the environmental conditions, the plant organ and the developmental stage. We performed our analysis in the juvenile growth stage on fully expanded leaves as easily accessible organ. Additionally, we tested for correlations between tolerance traits and metabolite or transcript levels measured under drought stress and control conditions to find markers that are independent of the water supply.

### The Test Population

For MAS, the correlation between marker and tolerance must hold in a wide range of genetic backgrounds. We therefore tested potential expression and metabolite markers in 21 rice cultivars. Most of the cultivars were selected from a Vietnamese breeding program to gain a test population comparable to the breeding material, for which the markers are intended. Our test population represented the two major subspecies of rice, *indica* and *japonica*. The substructure in our data set needs to be taken into account, as otherwise, like in association mapping, false-positive associations between genotype – or in our case marker - and phenotype may result [40]. For association mapping, statistical approaches are available to control the influence of the substructure [41]. In our study, cultivars belonging to the *indica* subspecies tended to be more tolerant than the *japonica* cultivars. Subspecies-specific differences in a metabolite level can thus lead to pseudocorrelations. The consequence of the substructure in the population is clearly visible in the PCA on the metabolite data set, where the third component separated *indica* and *japonica* cultivars. For the transcript data, the substructure was broken by the pre-selection of the candidate genes [36]. The expression of these genes in two sensitive (both *japonica*) and two tolerant cultivars (one *indica* and one *japonica*) was significantly affected by condition × tolerance group interaction. The risk of pseudocorrelations was therefore much lower for the selected candidate genes than for the metabolite markers.

The samples for the marker search were taken in the early vegetative growth phase of the cultivars before flower initiation. Under the climate chamber conditions employed in the experiment, the cultivar with the shortest live cycle (Nipponbare) flowered 55 days after sowing, most of the other cultivars flowered about 100 days after sowing, some considerably later (Köhl, unpublished data). By precise definition of the sampling time in the vegetative growth phase, we reduced the effect of differences in the live cycle term between cultivars on the validity of the marker. The cultivars showed considerable variation in height and tiller number (see accompanying paper Do et al. PLOS ONE 10.1371/journal.pone.0060325) and cultivars with short shoots generally grew more tillers than cultivars with high shoots. Thus, the selected population represented the variance in growth patterns found in rice.

### Multi-parallel Methods for Marker Search

Metabolite and expression profiling both allow multi-parallel measurements of several hundreds to thousands of parameters with predictive capability. Each method has their relative merits. Expression profiling by microarray hybridization is by now well established for several crops. Based on such analyses, PCR based tests can be established for candidate genes. If a linked genetic marker can be identified, a genome-based test can be designed. This allows fast screening at an early growth stage, independent of environmental conditions. If the functions of the proteins encoded by the genes showing altered expression are known, regulatory or metabolic pathways that affect drought tolerance can be identified. The gained insight into drought tolerance mechanisms can then be used to increase tolerance by altering the expression of a key gene [24], [42].

Metabolite profiling generally yields less response parameters than expression profiling and the ratio between found analytes and known metabolites is generally worse than the ratio between genes of known and unknown function. In rice, for which metabolite profiling is still in an early stage, GC-MS profiling yields 50–150 known metabolites [43]. In spite of this limitation, metabolomics is becoming a major tool to study plant stress responses [44]–[48] and will become a key factor in molecular breeding [49]–[51]. A major advantage of metabolite profiling is the huge body of reference data available from more than a hundred years of biochemical research compared to only thirty years of genomic research. If a metabolite is found to correlate with stress tolerance, relevant pathways, in which the metabolite is involved, can thus be rapidly identified and the mechanism of tolerance unravelled. In contrast to most genes, metabolite markers provide condensed information over several processes [52]. Thus, metabolite and gene expression markers both have their advantages.

The main disadvantage of metabolite and expression markers is their lower stability compared to genomic markers. Metabolite and RNA concentrations can be influenced by diurnal rhythm, environmental conditions and developmental stage of the plant. This can be taken into account by standardising the sampling conditions and choosing developmental stages that are metabolically relatively stable (e.g. vegetative growth in Poaceae) and time intervals in the diurnal cycle, in which metabolite and transcript concentrations change but slowly [53]. Another approach is to choose metabolite or transcript markers, in which concentration differences between tolerant and sensitive cultivars are large compared to the changes caused by environmental factors or diurnal rhythms. In contrast to transcript and metabolite markers, genomic DNA markers like SSR or SNPs are independent of environmental conditions and developmental stages. The identification of genomic markers by association or QTL mapping requires phenotyping and genotyping of a sufficiently large segregating mapping population [54] and is thus much more labour-intensive than the identification of metabolite or transcript markers. However, both approaches can be combined. Instead of doing a genome-wide association study, the region of interest can be narrowed down to the location of candidate genes from transcript profiling. In contrast to genomic markers, transcript and metabolite markers can be preselected based on their response to the stress, for which tolerance markers are to be identified.

### Marker Identification by Correlation Analysis

To test the value of potential markers, we first characterised a population of 21 rice cultivars for drought tolerance and phenotyped them for traits that had been used to predict drought tolerance. Levels of 46 candidate genes and 79 metabolites were measured in leaves of 21 cultivars, which had been grown under control and drought conditions. Potential drought tolerance markers were identified by analysing correlations of expression and metabolite levels with the phenotypic traits. Significant positive correlations of metabolite or transcript levels with phenotypic traits indicate a high expression or metabolite level in tolerant cultivars, while negative correlations indicate high levels in sensitive cultivars. As high levels of a metabolite or transcript can be ascertained more reliably than low levels or absence, we focus on the prediction from high levels. In the first case (positive correlation), the metabolite or gene expression would be a tolerance marker, as high levels indicate tolerance. Gene expression or metabolites with negative correlation are sensitivity markers.

The accumulation of a metabolite or transcript under stress is not necessarily functionally connected with an increase in the tolerance level or with tolerance differences between genotypes. Metabolite levels can increase as a result from an accelerated degradation or a reduced biosynthesis of another metabolite without any protective effect. Likewise, not all candidate genes, for which expression levels are significantly correlated with physiological data, are necessarily connected with drought tolerance. It could, for example, also be generally correlated with the growth rate not only under drought but also under control conditions. To identify such false positives, we studied the correlation between expression and metabolite levels under control conditions and phenotypic traits measured under these conditions. Indeed, expression levels of, for example, the genes coding for an asparagine synthetase and an AMP deaminase were negatively correlated with shoot and total fresh and dry weight under drought conditions as well as under control conditions. This suggests that high expression of those genes indicated slow growth rather than specific performance under drought.

### Metabolite Markers

Among metabolite levels, potential sensitivity markers were identified that correlated significantly and negatively with phenotypic traits under drought stress. This group contains many amino acids (asparagine, glutamate, glutamine, glycine, serine and threonine). Their concentrations were high in drought-sensitive cultivars with low biomass under drought stress. This agrees with findings in *Arabidopsis thaliana* grown under optimal conditions where intermediates of the central metabolism were mostly negatively correlated with biomass production [48], [55]. High amino acid levels observed in sensitive cultivars reflect the increase in protein degradation and the decrease in protein synthesis under drought stress [36], [48], [56], [57]. Accordingly, high amino acid levels have been observed in plants subjected to other stresses and in senescing leaves [58]–[61].

In contrast, eight metabolites were identified, whose levels under drought stress were higher in tolerant than in sensitive cultivars. This pattern was found for allantoin, galactaric and gluconic acid, glucose and salicylic acid glucopyranoside plus three unidentified metabolites. These metabolites are promising candidates for drought tolerance markers. Especially mid-day glucose level in young leaves is an interesting marker candidate, as levels of glucose were already shown to be increased during drought in Eucalyptus [61], [62], during salt stress in Lotus [48] and to correlate significantly with acclimated freezing tolerance in *Arabidopsis thaliana* [48], [63], [64]. In all these stresses, glucose may be part of a C-based osmotic adjustment [65], [66]. In contrast to sucrose and starch, glucose concentration is not negatively correlated with biomass production under unstressed conditions [67]. The positive correlations were restricted to plants under stress conditions and were not found under control conditions. Thus, no constitutive metabolite markers could be identified.

### Gene Expression Markers

Among the 46 candidate genes selected from a previous study [36], more than 20% showed significant correlation between expression and plant performance under drought stress in the test population. Similar to the metabolite markers, negative correlations dominated. For many of these sensitivity markers, the correlation with performance under drought could also be found for expression levels measured under control conditions. These sensitivity markers seem to be constitutive and may thus be useful to exclude germplasms from a breeding population at an early stage. In the case of asparagine synthetase, the increased gene expression in sensitive cultivars was matched by an increased asparagine level in these cultivars.

Many of the genes with a positive correlation between expression and performance under drought showed this correlation exclusively under drought conditions. These genes are scientifically interesting, but of limited value as breeding markers. These tolerance markers code for proteins involved in several pathways, which fits the general assumption of a multigenic nature of drought tolerance. Remarkably, expression levels of a cytosolic (cFBPase) and a plastidial fructose-1,6-bisphosphatase (pFBPase) were positively correlated with drought tolerance or photosynthetic yield under drought stress, respectively. In sensitive cultivars, the cFBPase was slightly down-regulated under drought stress, whereas it was significantly induced in tolerant cultivars [36]. Both enzymes are physiologically and biochemically very well studied [68]–[70]. The plastidial enzyme catalyzes a rate-limiting, highly regulated step in the Calvin-Benson cycle [71] towards regeneration of ribulose-bis-phosphate and starch production in the chloroplast. The cytosolic enzyme promotes a highly regulated step in the conversion of triose phosphates into sucrose, which then may be exported to sink organs such as roots. A down-regulation of the genes for the cytosolic enzyme has been observed before in water-stressed sunflower plants [72]. The authors suggest that the down-regulation of this enzyme could be involved in the non-stomatal limitation of photosynthesis [72]. Additionally, it is discussed that rates of sucrose synthesis and rates of photosynthesis may be coordinated by changes in the activity of this enzyme [73]. In the Arabidopsis mutant (*hcef* ) with increased cyclic electron flow around photosystem I, the mutation has been mapped to the pFBPase [74]. Antisense repression of cFBPase reduced sucrose synthesis in Arabidopsis [75] and potato [76]. When cFBPase is overexpressed together with the triose phosphate/phosphate transporter, photosynthetic CO_2_ assimilation rates are enhanced and glucose levels increased compared to the wildtype [77]. The increased expression of both *FBPase* genes in tolerant rice cultivars under stress is counterintuitive, as the enzymes are crucial parts of competing pathways. However, a similar situation was revealed in a detailed metabolic analysis of Arabidopsis under drought stress [65]. The activity of AGPase, the rate limiting enzyme in plastidial starch synthesis, was increased in severely drought-stressed plants compared to control plants during the entire diurnal cycle. At the same time, the amount of sucrose exported to the roots increased under drought stress. A possible explanation is that a higher expression level of both genes may allow a higher turnover of the enzymes and thus an increased regulatory capacity for the switch between photosynthetic CO_2_ fixation and starch storage in the chloroplasts and carbohydrate exports to the sinks, especially the roots. The adaptive value of modifications in the source/sink relationship has been shown in rice. Increase in cytokinin synthesis by genetic modification improved grain yield under drought [78]. A regulation switch with a high capacity is obviously only needed, if triose phosphates are produced by photosynthesis. In sensitive cultivars, where chlorosis and necrosis reduced photosynthesis under drought stress, this regulatory capacity may not be needed, thus gene expression levels may be decreased.

Altogether, the application of expression and metabolic profiling methods on rice cultivars subjected to long-term drought stress revealed several marker candidates for drought tolerance. The most promising markers were glucose, high levels of which indicated high tolerance and high expression levels of the *cFBPase* and *pFBPase* genes. Their elevated expression in tolerant cultivars may contribute to the adjustment of photosynthesis and source-sink relationships under drought. The test population, in which these marker candidates were identified, was highly diverse in drought tolerance and genetic background. This makes it likely that the markers are useful for breeder’s selection in a wide range of rice germplasm. As the correlations between transcript and metabolite levels and drought tolerance were found in a controlled-environment drought stress system, the next step required would be the validation in field experiments. These experiments would also give insight into the effect of environmental factors other than water supply on the potential markers.

## Materials and Methods

### Plant Material and Stress Treatment

Twenty-one rice (*Oryza sativa* L.) cultivars (Table 1) originating either from the IBT (Institute of Biotechnology, Hanoi, Vietnam) or from the IRRI (International Rice Research Institute, Manila, Philippines) were grown under water sufficient and water limiting conditions in three independent experiments (#1–3) in a controlled climate chamber as described by Degenkolbe et al. [36]. The design was a split-plot design with five blocks per drought or control treatment. Each treatment and cultivar was represented by five replicate pots with one plant per pot. Pots were randomized within the blocks. Block position was rotated daily. Plants were cultivated in 10 cm pots on a 7.5 cm deep layer of an artificial substrate. The shallow substrate level was chosen to reduce the effect of differences in rooting patterns between cultivars on the result. Pots were positioned in polypropylene boxes filled with water to the level of the substrate surface. Rice plants were grown in 12 h day (600 µE m^−2^ s^−1^) with 26°C and 75% relative humidity in the light and 22°C and 70% relative humidity at night. Twenty-six days after sowing, water was removed from half of the boxes and plants were left to dry for four days, until the soil water content had reached the permanent wilting point (PWP) for 50% of the plants. Thereafter, the soil water content was kept constant to the fixed PWP value over a period of 14 days by weighing each pot at the end of the light period and adding the amount of water lost during the last 24 hours.

After 18 days of drought stress, plants were harvested four to six hours after the beginning of the light period. Samples for expression and metabolic profiling were harvested from the middle section of the blades of fully expanded green leaves, weighted and immediately frozen in liquid nitrogen, and stored at -80°C until use.

Cultivars were genotyped for seven subspecies-specific sequence tagged site (SS-STS) markers [37] as described before [36].

### RNA Isolation and cDNA Synthesis

Frozen leaf material was homogenized in a ball mill for 90 sec at 28 Hz. Plant material from five replicate plants per cultivar and condition was pooled and 60 to 90 mg were used for total RNA isolation using the NucleoSpin RNA plant kit (Macherey-Nagel, Düren, Germany) following manufacturer’s instruction. RNA concentration was determined with the Nanodrop N-1000 Spectrophotometer (Nanodrop Technologies, Wilmington, DE). To remove remaining genomic DNA, samples were treated with Baseline-ZERO DNase. The absence of genomic DNA contamination was subsequently confirmed by quantitative RT-PCR (qRT-PCR) with primers for an intron (LOC_Os01g01840). The integrity of total RNA was checked on an 1.7% (w/v) agarose gel. cDNA synthesis from 4 µg of total RNA with Superscript III reverse transcriptase was performed following the manufacturer’s instruction (Invitrogen, Karlsruhe, Germany). Quality of synthesized cDNA was checked by qRT-PCR with two primer pairs binding to the 3′ and 5′ ends of the actin 1 (LOC_Os03g50890) transcript, respectively.

### Quantitative RT-PCR

Expression levels of 46 candidate genes (Table S1) were measured in leaf material from 21 rice cultivars grown under control and drought stress conditions in three experiments. Primers for qRT-PCR (Table S4) were designed on the published *japonica* sequence with PrimerExpress 2.0 (Applied Biosystems, Darmstadt, Germany) and checked with NetPrimer (www.premierbiosoft.com/netprimer/netprlaunch/netprlaunch.html). Sequences were blasted on the databases of GRAMENE (www.gramene.org) and the Beijing Genomics Institute to ensure specific amplification in both *japonica* and *indica* cultivars. Correct size of the amplified region for each primer pair was checked by agarose gel electrophoresis.

qRT-PCR was performed with the ABI Prism 7900HT (Applied Biosystems, Foster City, CA) using SYBR Green Master Mix (Eurogentec, Köln, Germany) with standard thermal cycling conditions (50°C for 2 min, 95°C for 10 min, 40 cycles of 95°C for 15 sec and 60°C for 1 min). Dissociation curves were checked with the SDS 2.2.1 software (Applied Biosystems) for shoulders or additional peaks. The expression values were normalised to the expression of the housekeeping genes actin 1 and cyclophilin and the primer efficiency as described before [36]: ‘Normalised expression of the genes of interest was calculated by dividing the average relative expression (primer efficiency P to the power of cycle number Ct) of the two housekeeping genes (H1 and H2) by the relative expression of the gene of interest (GOI): ((P_H1_∧Ct_H1_+ P_H2_∧Ct_H2_)/2)/P_GOI_∧Ct_GOI_. Primer efficiency was calculated using LinRegPCR [79].

### GC-ToF-MS

From 120 mg of frozen, ground leaf material from experiment #1 and 2, a fraction enriched in polar primary metabolites was prepared and processed as described previously [80]. Gas chromatography coupled to electron impact ionization-time of flight-mass spectrometry (GC/EI-TOF-MS) was performed on an Agilent 6890N24 gas chromatograph attached to a Pegasus III mass spectrometer, LECO, St. Joseph, USA [81]. Chromatograms were pre-processed with ChromaTOF software 1.00, Pegasus driver 1.61 (Leco; http://www.leco.de). Selective peak heights representing arbitrary mass spectral ion currents were normalised by sample dry weight and to an internal standard that was added upon extraction of the polar metabolite fraction. Data were subsequently processed with TagFinder [82]. Analytes that were detected in less than 50% of control and 50% of drought-stressed plants were excluded from the dataset. Clusters of at least three corresponding mass fragments were selected for relative metabolite quantification. Metabolites were identified by matching to references in the Golm Metabolome Database [83]. The matching process was manually supervised for a match factor >500 and retention index deviations <1% [84].

Outlier samples were detected in plots of a principal component analysis (PCA; R package pcaMethods; [80], [85] of raw data and were removed from further analysis. Log-transformed metabolite levels were normalised by subtracting the median metabolite level for each experiment and metabolite to remove the effect of experiment and GC-MS run. Mean values of normalised metabolite levels were calculated for each cultivar, condition and experiment and analysed by PCA with the settings mean centred matrix and unit variance scale (R package pcaMethods). Euclidean distance of scaled data was used for hierarchical clustering.

### Correlation Analysis

Expression of candidate genes and metabolites levels were analysed for Pearson correlations (cor.test function, R) with physiological data that are indicative of drought tolerance, namely shoot and total fresh and dry weight, mean scoring rank, mean water use efficiency and photosynthesis yield. Expression and metabolite data were log-transformed. Scoring ranks were multiplied with -1. The correlation analysis was performed on three variable combinations, namely correlating (1) expression/metabolite data from drought-stressed plants with performance parameters from drought-stressed plants, (2) expression/metabolite data from control plants with performance parameters under stress and (3) expression/metabolite data from control plants with performance parameters under control conditions.

## Supporting Information

Figure S1 **Average shoot dry weight in rice cultivars plotted against the relative expression of 46 genes.** The regression coefficient r for the linear regression of shoot dry weight against expression level is shown in the upper left corner. The primer number and the gene name are indicated in the title of each figure. The complete name for each gene can be retrieved from Table S4. File SupportingFigure2.pdf, Format pdf.
(PDF)

Figure S2 **Average shoot dry weight in rice cultivars plotted against the level ( = signal intensity) of 79 metabolites.** The regression coefficient r for the linear regression of shoot dry weight against metabolite level is shown in the upper left corner. Unidentified metabolites are labelled with their retention time index MST. File SupportingFigureS4.pdf. Format pdf.
(PDF)

Table S1 **Mean normalised expression values for 45 genes measured in 21 cultivars under drought and control conditions.** Cultivar identifiers see manuscript Table 1. Condition d = drought, c = control. Primer identifiers indicated in column heading see Table S4. File Supplemental TableS1.xls, Format xls.
(XLS)

Table S2 **Mean normalised metabolite levels measured in rice cultivars under drought and control conditions.** Cultivar identifiers see Table S1. Condition d = drought, c = control. Metabolite identifiers indicated in column heading see Table S3. NA = not detected. File Supplemental Table S3.xls, Format xls.
(XLS)

Table S3 **Metabolite identifiers (Mid), retention times, metabolite names and loadings of the first five principal components.** File Supplemental Table S5.pdf, Format pdf.
(PDF)

Table S4 **List of qPCR primer sequences that were used for quality checks and RT-PCR together with their Primer Identifier (PId), TIGR Locus identifier (Locus ID), OligoID from the gene chip (see Degenkolbe et al. 2009), and direction (FW = forward, RV = reverse).** File Supplemental Table S6.pdf, Format pdf.
(PDF)
