# Supplementary material for: Identification of Drought Tolerance Markers in a Diverse Population of Rice Cultivars by Expression and Metabolite Profiling
Source: PLoS One. 2013 May 22;8(5):e63637. doi: 10.1371/journal.pone.0063637 (PMC3661581; doi:10.1371/journal.pone.0063637)
Supplement: Figure S1 — Average shoot dry weight in rice cultivars plotted against the relative expression of 46 genes. The regression coefficient r for the linear regression of shoot dry weight against expression level is shown in the upper left corner. The primer number and the gene name are indicated in the title of each figure. The complete name for each gene can be retrieved from Table S4. File SupportingFigure2.pdf, Format pdf. (PDF) [file pone.0063637.s001.pdf]

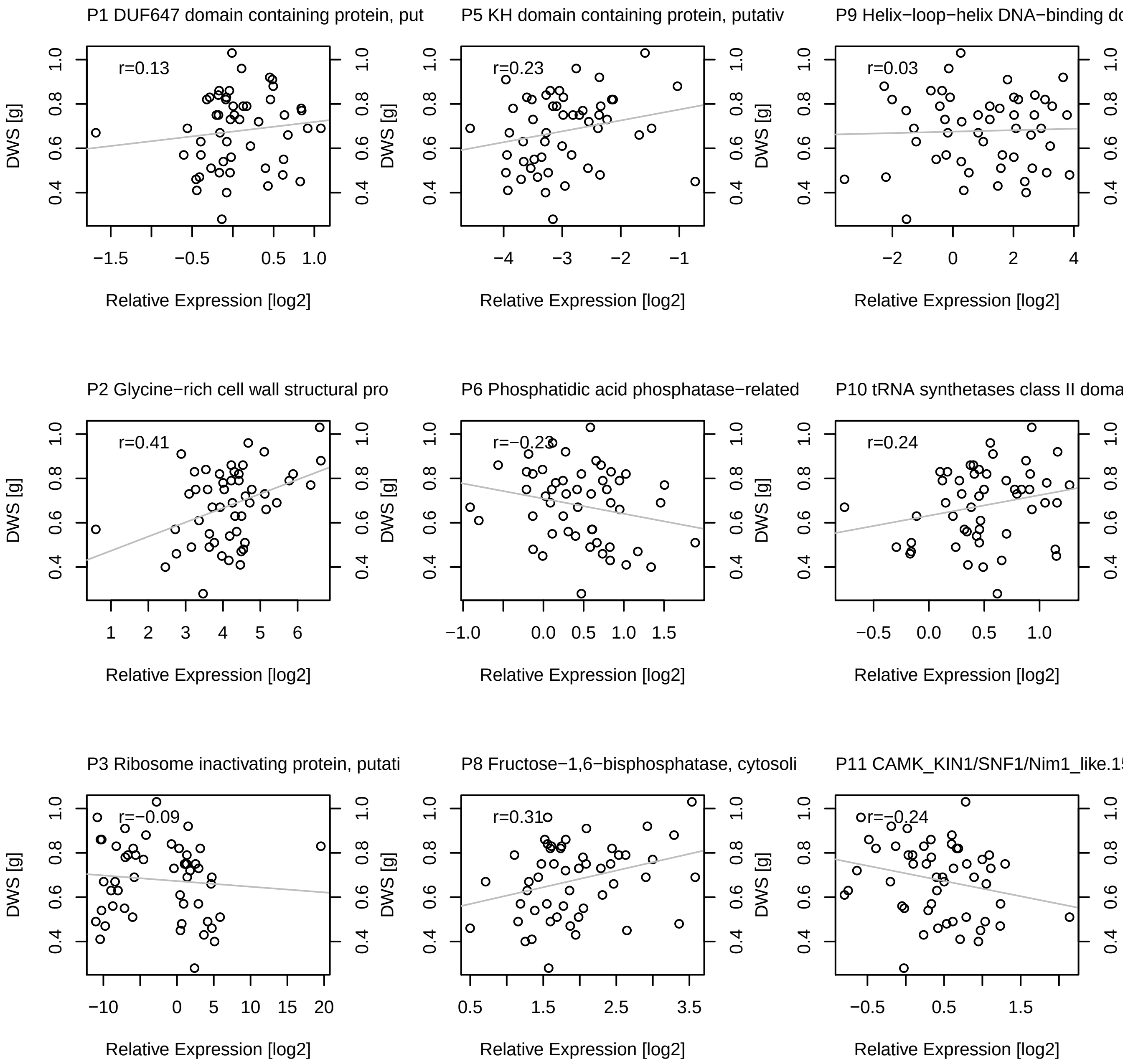

P1 DUF647 domain containing protein, put
r=0.13
P5 KH domain containing protein, putativ
r=0.23
P9 Helix−loop−helix DNA−binding do
r=0.03
P2 Glycine−rich cell wall structural pro
r=0.41
P6 Phosphatidic acid phosphatase−related
r=−0.23
P10 tRNA synthetases class II doma
r=0.24
P3 Ribosome inactivating protein, putati
r=−0.09
P8 Fructose−1,6−bisphosphatase, cytosoli
r=0.31
P11 CAMK_KIN1/SNF1/Nim1_like.1
r=−0.24
DWS [g]
Relative Expression [log2]

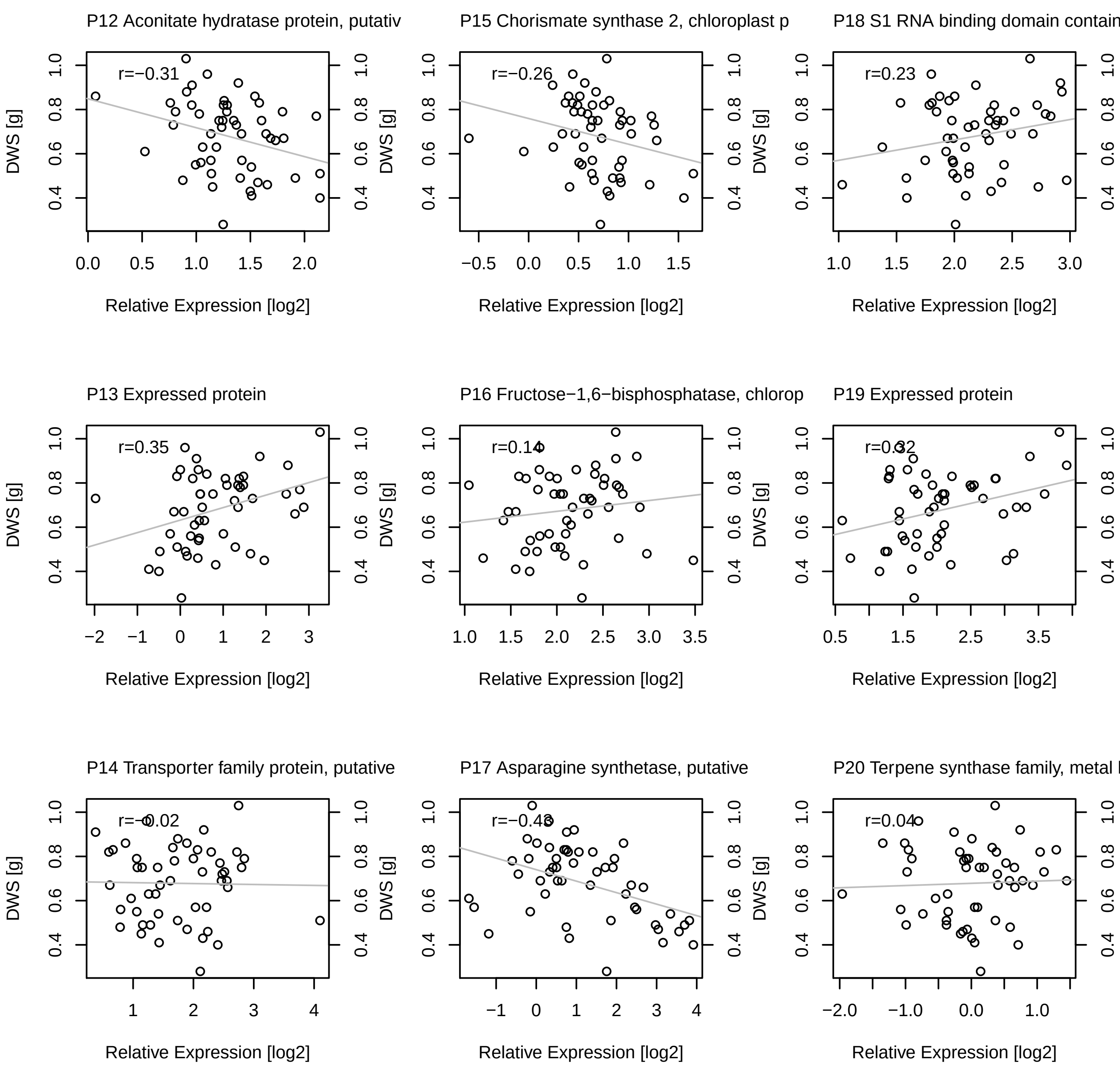

P12 Aconitate hydratase protein, putativ
r=−0.31
P15 Chorismate synthase 2, chloroplast p
r=−0.26
P18 S1 RNA binding domain contain
r=0.23
P13 Expressed protein
r=0.35
P16 Fructose−1,6−bisphosphatase, chlorop
r=0.14
P19 Expressed protein
r=0.32
P14 Transporter family protein, putative
r=−0.02
P17 Asparagine synthetase, putative
r=−0.43
P20 Terpene synthase family, metal b
r=0.04
DWS [g]
Relative Expression [log2]

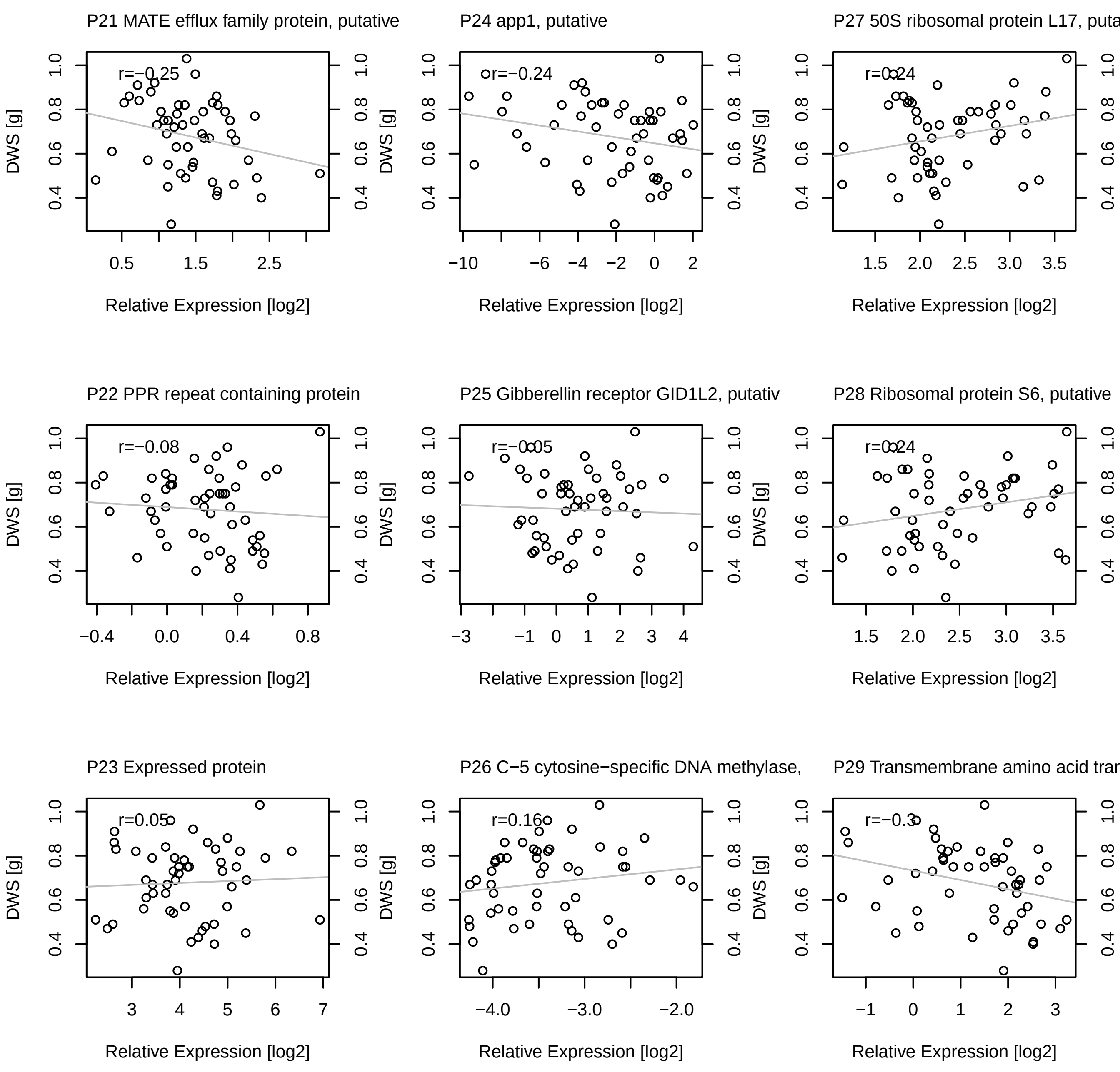

P21 MATE efflux family protein, putative
r=−0.25
P24 app1, putative
r=−0.24
P27 50S ribosomal protein L17, puta
r=0.24
P22 PPR repeat containing protein
r=−0.08
P25 Gibberellin receptor GID1L2, putativ
r=−0.05
P28 Ribosomal protein S6, putative
r=0.24
P23 Expressed protein
r=0.05
P26 C−5 cytosine−specific DNA methylase,
r=0.16
P29 Transmembrane amino acid tran
r=−0.3
DWS [g]
Relative Expression [log2]

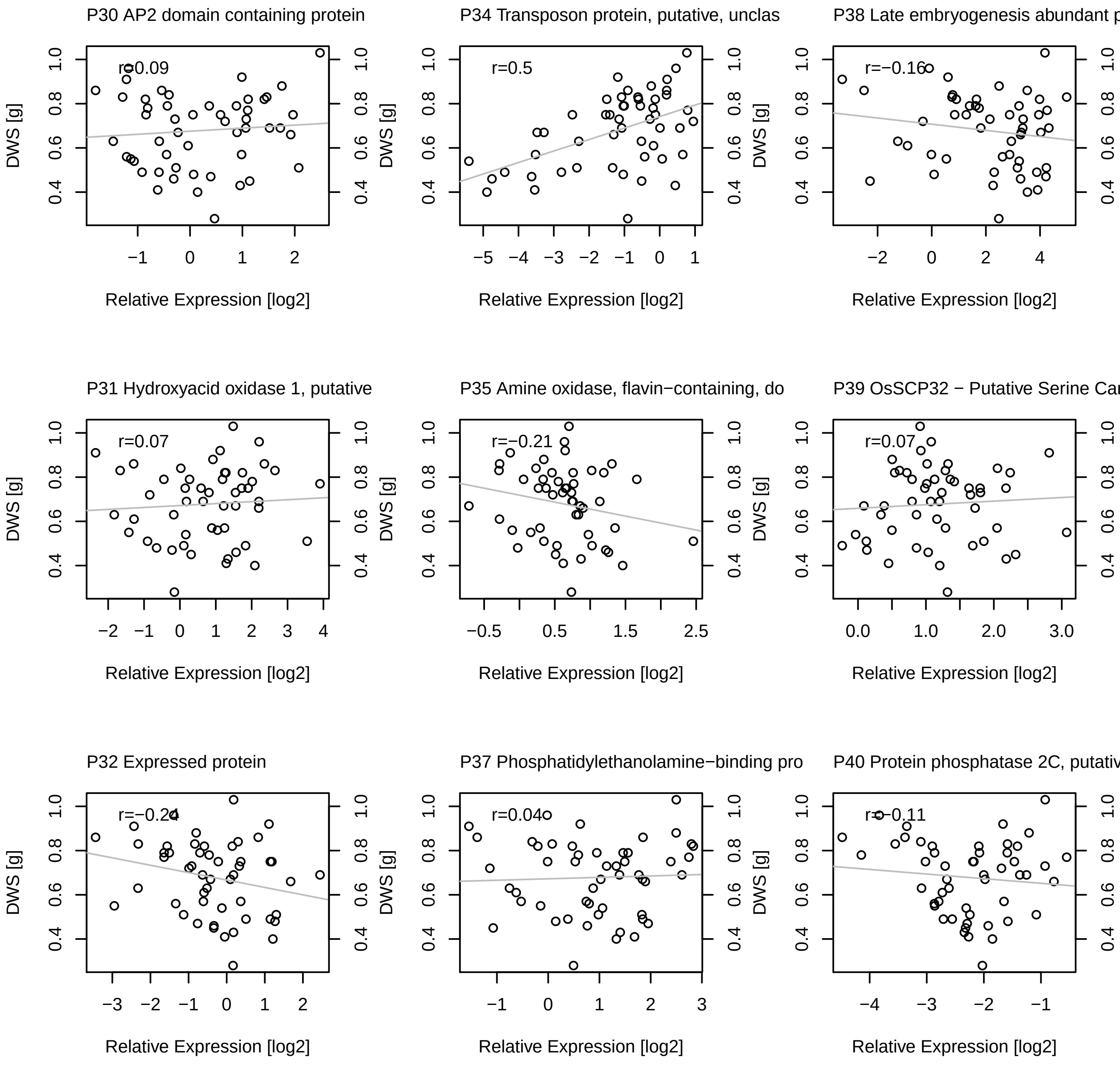
P30 AP2 domain containing protein
r=0.09
P34 Transposon protein, putative, unclas
r=0.5
P38 Late embryogenesis abundant p
r=−0.16
P31 Hydroxyacid oxidase 1, putative
r=0.07
P35 Amine oxidase, flavin−containing, do
r=−0.21
P39 OsSCP32 − Putative Serine Car
r=0.07
P32 Expressed protein
r=−0.24
P37 Phosphatidylethanolamine−binding pro
r=0.04
P40 Protein phosphatase 2C, putativ
r=−0.11
DWS [g]
Relative Expression [log2]

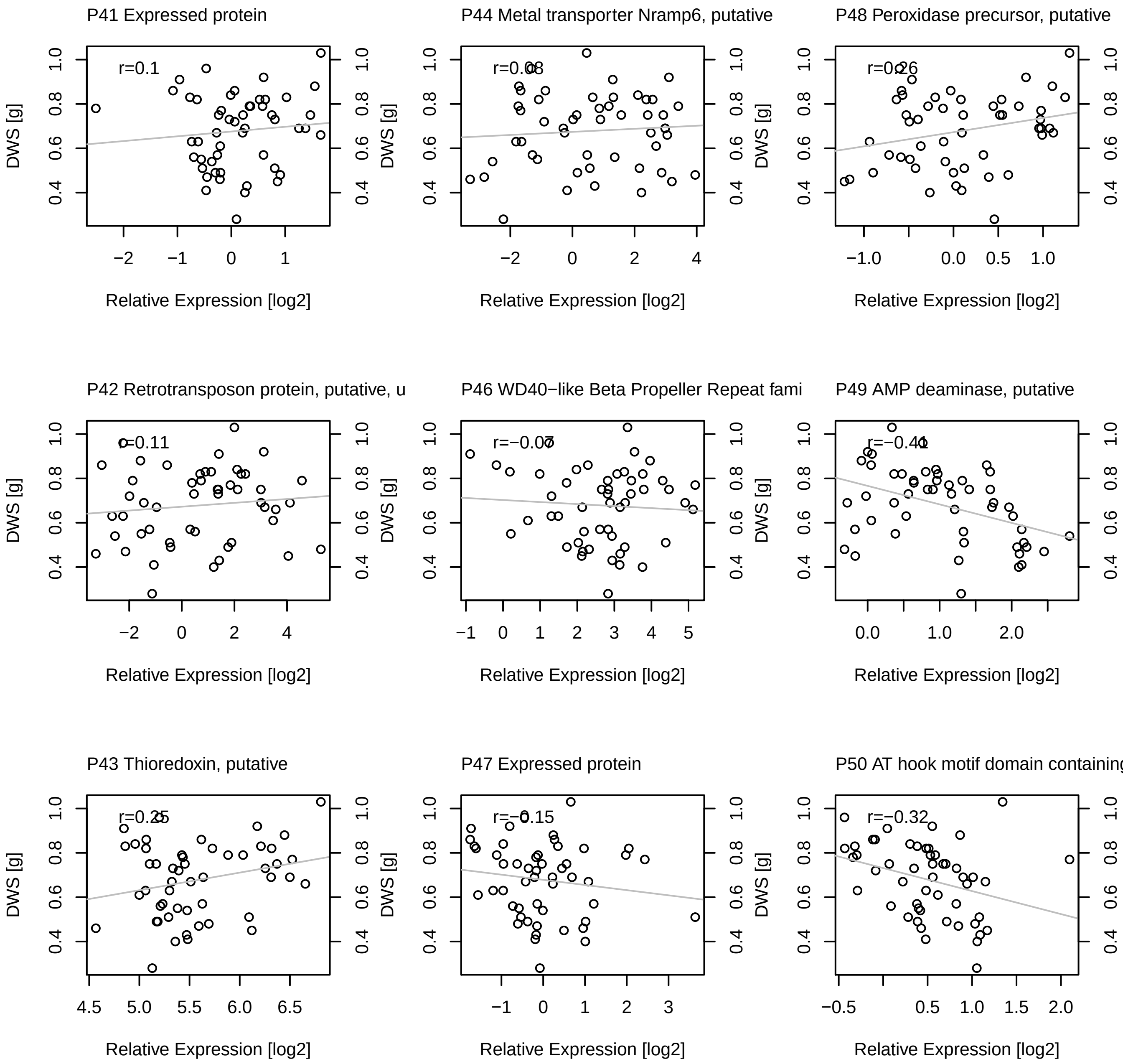

P41 Expressed protein
r=0.1
P44 Metal transporter Nramp6, putative
r=0.08
P48 Peroxidase precursor, putative
r=0.26
P42 Retrotransposon protein, putative, u
r=0.11
P46 WD40−like Beta Propeller Repeat fami
r=−0.07
P49 AMP deaminase, putative
r=−0.41
P43 Thioredoxin, putative
r=0.25
P47 Expressed protein
r=−0.15
P50 AT hook motif domain containing
r=−0.32
DWS [g]
Relative Expression [log2]

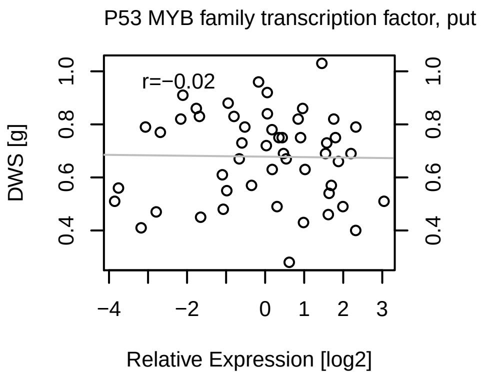

P53 MYB family transcription factor, put
r=−0.02
DWS [g]
0.4
0.6
0.8
1.0
−4
−2
0
1
2
3
Relative Expression [log2]
