# Supplementary material for: Identification of Drought Tolerance Markers in a Diverse Population of Rice Cultivars by Expression and Metabolite Profiling
Source: PLoS One. 2013 May 22;8(5):e63637. doi: 10.1371/journal.pone.0063637 (PMC3661581; doi:10.1371/journal.pone.0063637)
Supplement: Figure S2 — Average shoot dry weight in rice cultivars plotted against the level ( = signal intensity) of 79 metabolites. The regression coefficient r for the linear regression of shoot dry weight against metabolite level is shown in the upper left corner. Unidentified metabolites are labelled with their retention time index MST. File SupportingFigureS4.pdf. Format pdf. (PDF) [file pone.0063637.s002.pdf]

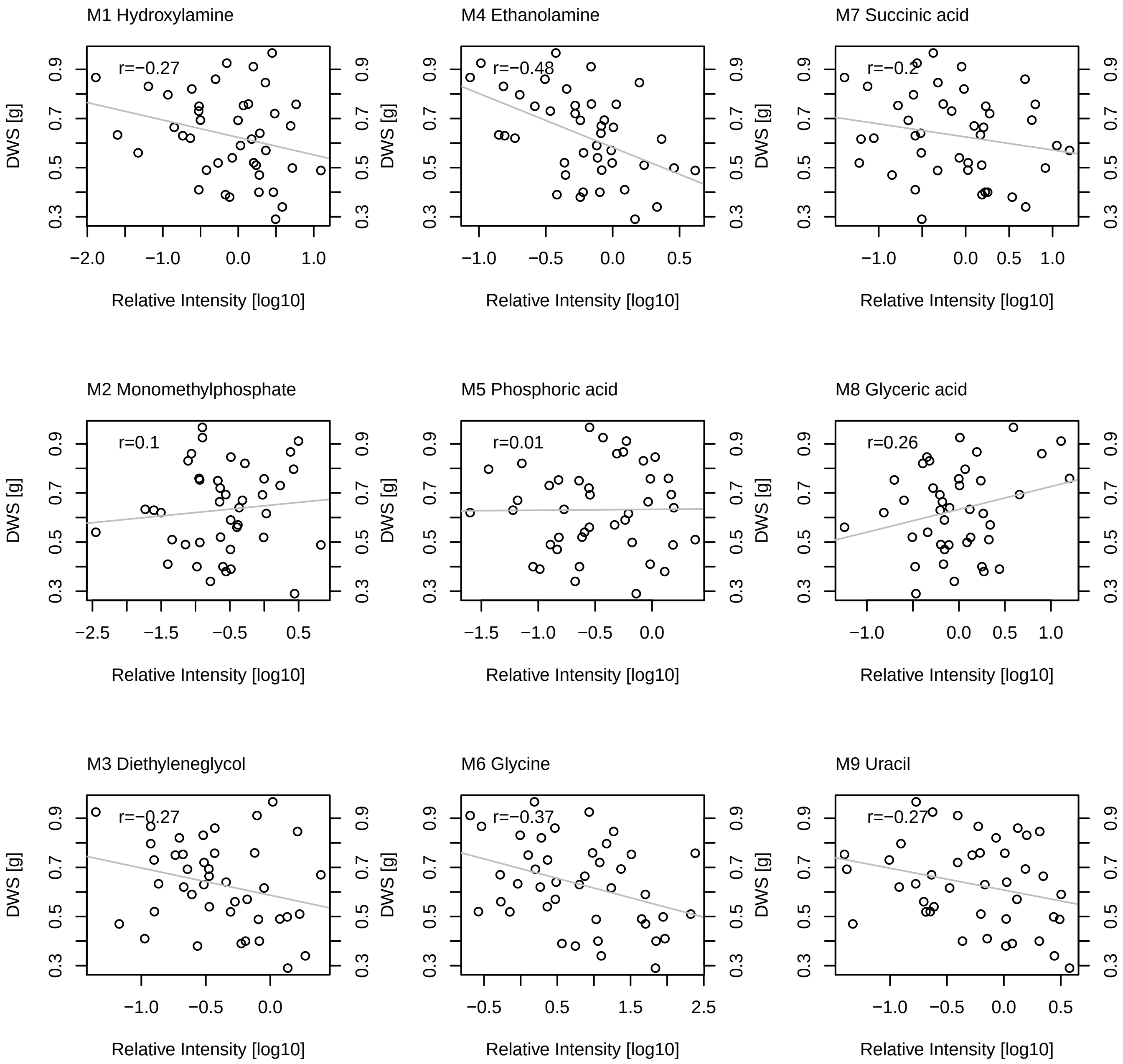
M1 Hydroxylamine
r=−0.27
M4 Ethanolamine
r=−0.48
M7 Succinic acid
r=−0.2
M2 Monomethylphosphate
r=0.1
M5 Phosphoric acid
r=0.01
M8 Glyceric acid
r=0.26
M3 Diethyleneglycol
r=−0.27
M6 Glycine
r=−0.37
M9 Uracil
r=−0.27
DWS [g]
Relative Intensity [log10]

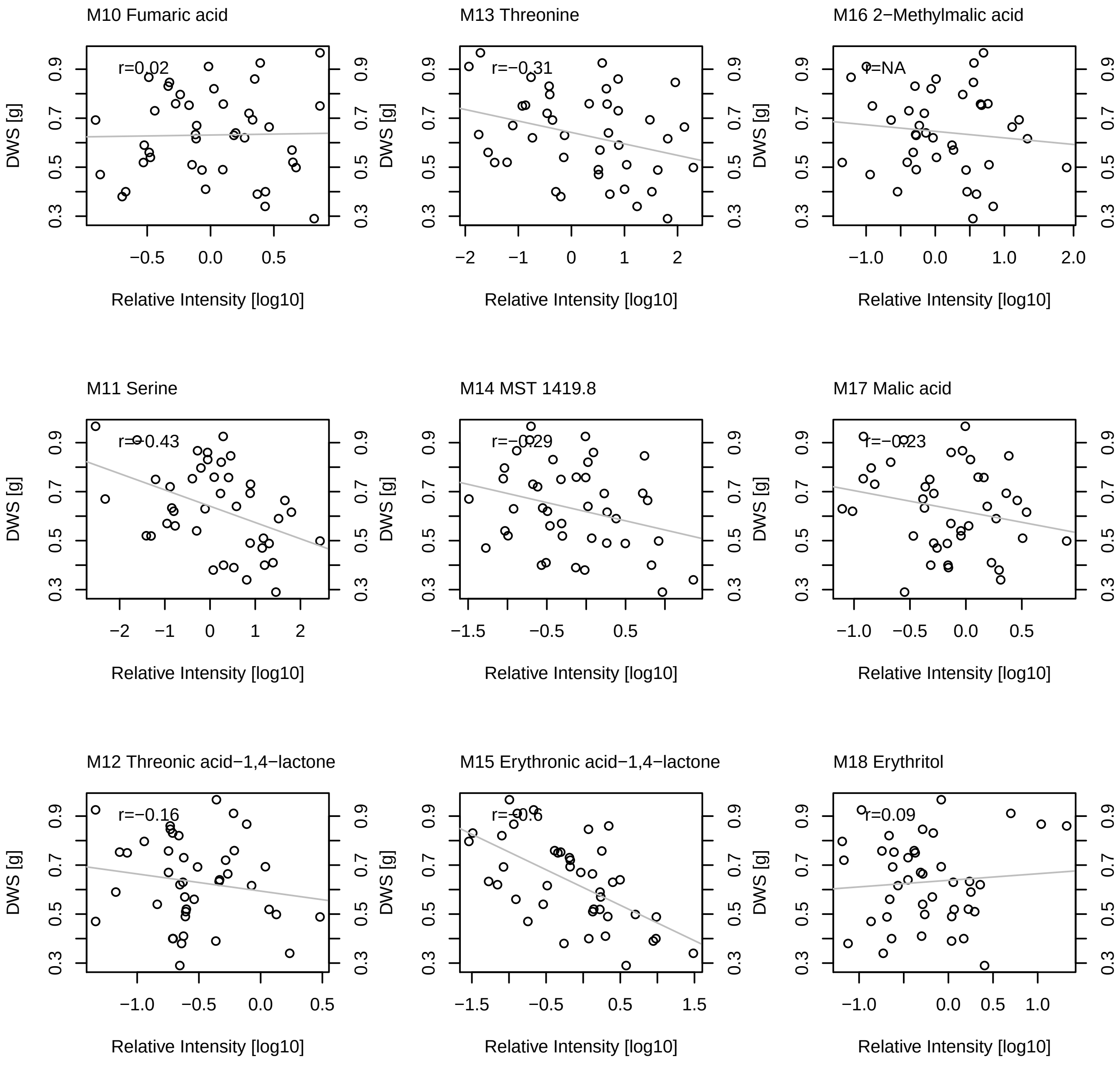
M10 Fumaric acid
r=0.02
M13 Threonine
r=−0.31
M16 2−Methylmalic acid
r=NA
M11 Serine
r=−0.43
M14 MST 1419.8
r=−0.29
M17 Malic acid
r=−0.23
M12 Threonic acid−1,4−lactone
r=−0.16
M15 Erythronic acid−1,4−lactone
r=−0.6
M18 Erythritol
r=0.09
DWS [g]
Relative Intensity [log10]

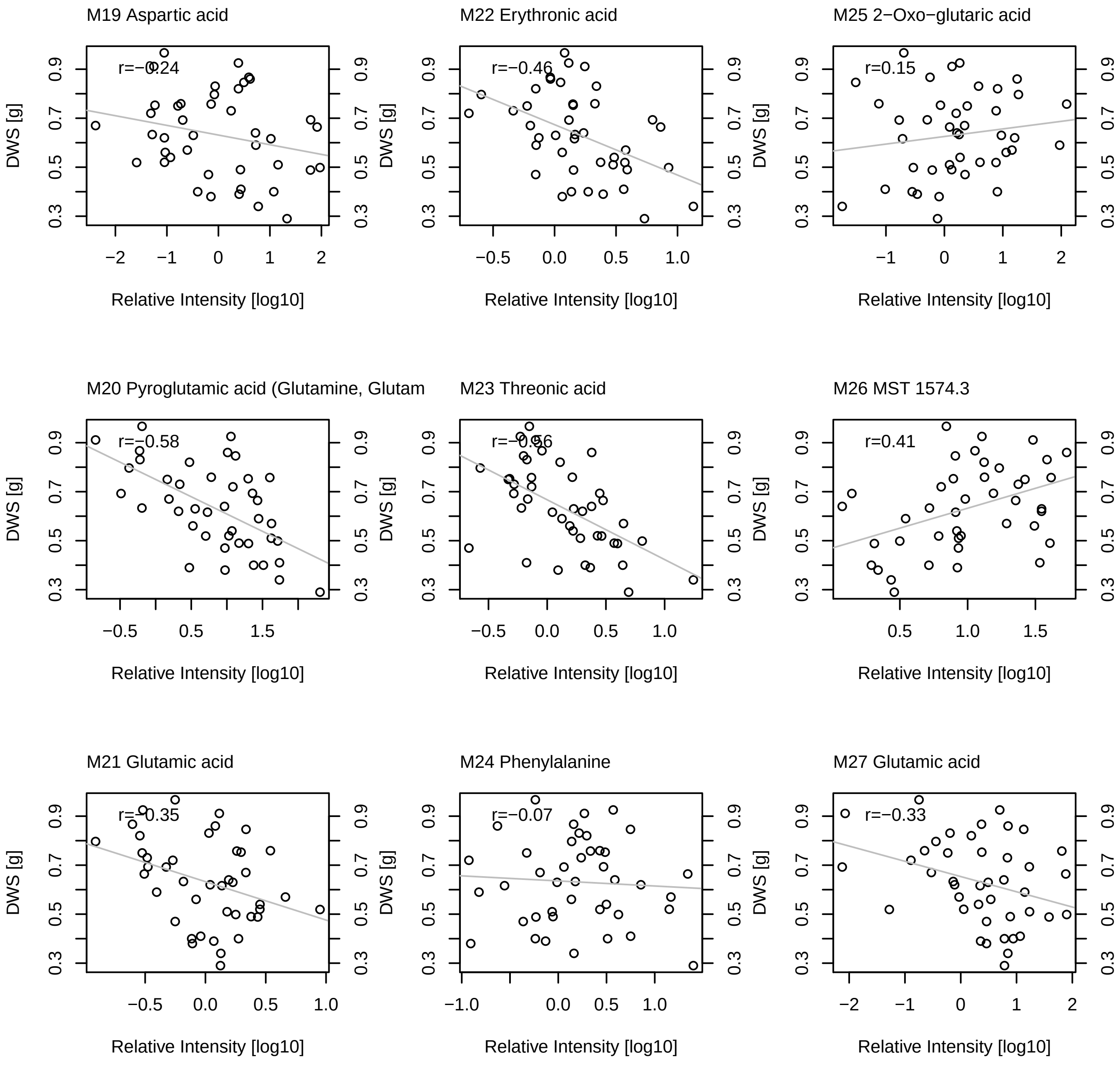
M19 Aspartic acid
r=−0.24
M22 Erythronic acid
r=−0.46
M25 2−Oxo−glutaric acid
r=0.15
M20 Pyroglutamic acid (Glutamine, Glutam
r=−0.58
M23 Threonic acid
r=−0.56
M26 MST 1574.3
r=0.41
M21 Glutamic acid
r=−0.35
M24 Phenylalanine
r=−0.07
M27 Glutamic acid
r=−0.33
DWS [g]
Relative Intensity [log10]

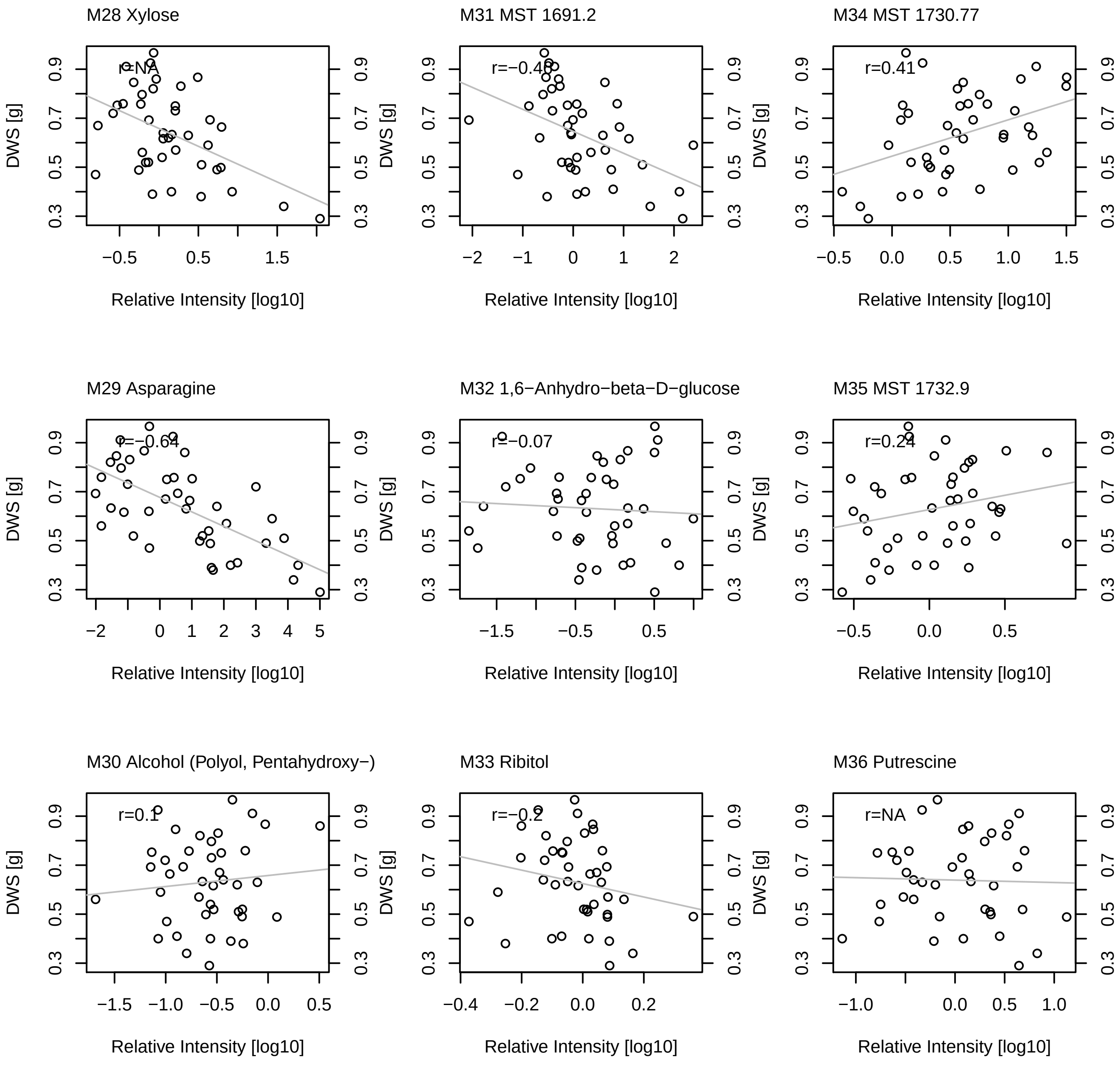
M28 Xylose
r=NA
M31 MST 1691.2
r=−0.48
M34 MST 1730.77
r=0.41
M29 Asparagine
r=−0.64
M32 1,6−Anhydro−beta−D−glucose
r=−0.07
M35 MST 1732.9
r=0.24
M30 Alcohol (Polyol, Pentahydroxy−)
r=0.1
M33 Ribitol
r=−0.2
M36 Putrescine
r=NA
DWS [g]
Relative Intensity [log10]

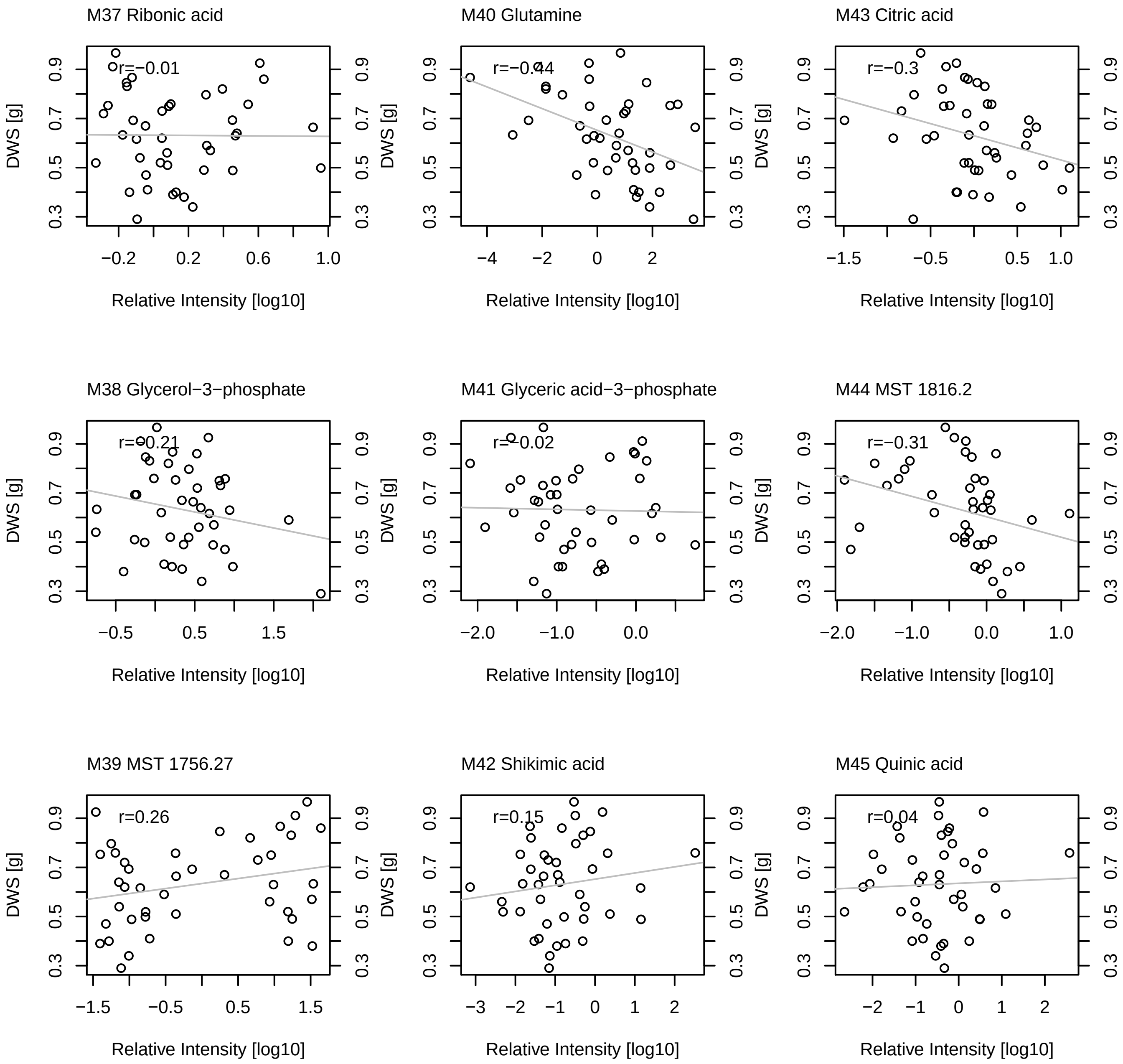
M37 Ribonic acid
r=−0.01
M40 Glutamine
r=−0.44
M43 Citric acid
r=−0.3
M38 Glycerol−3−phosphate
r=−0.21
M41 Glyceric acid−3−phosphate
r=−0.02
M44 MST 1816.2
r=−0.31
M39 MST 1756.27
r=0.26
M42 Shikimic acid
r=0.15
M45 Quinic acid
r=0.04
DWS [g]
Relative Intensity [log10]

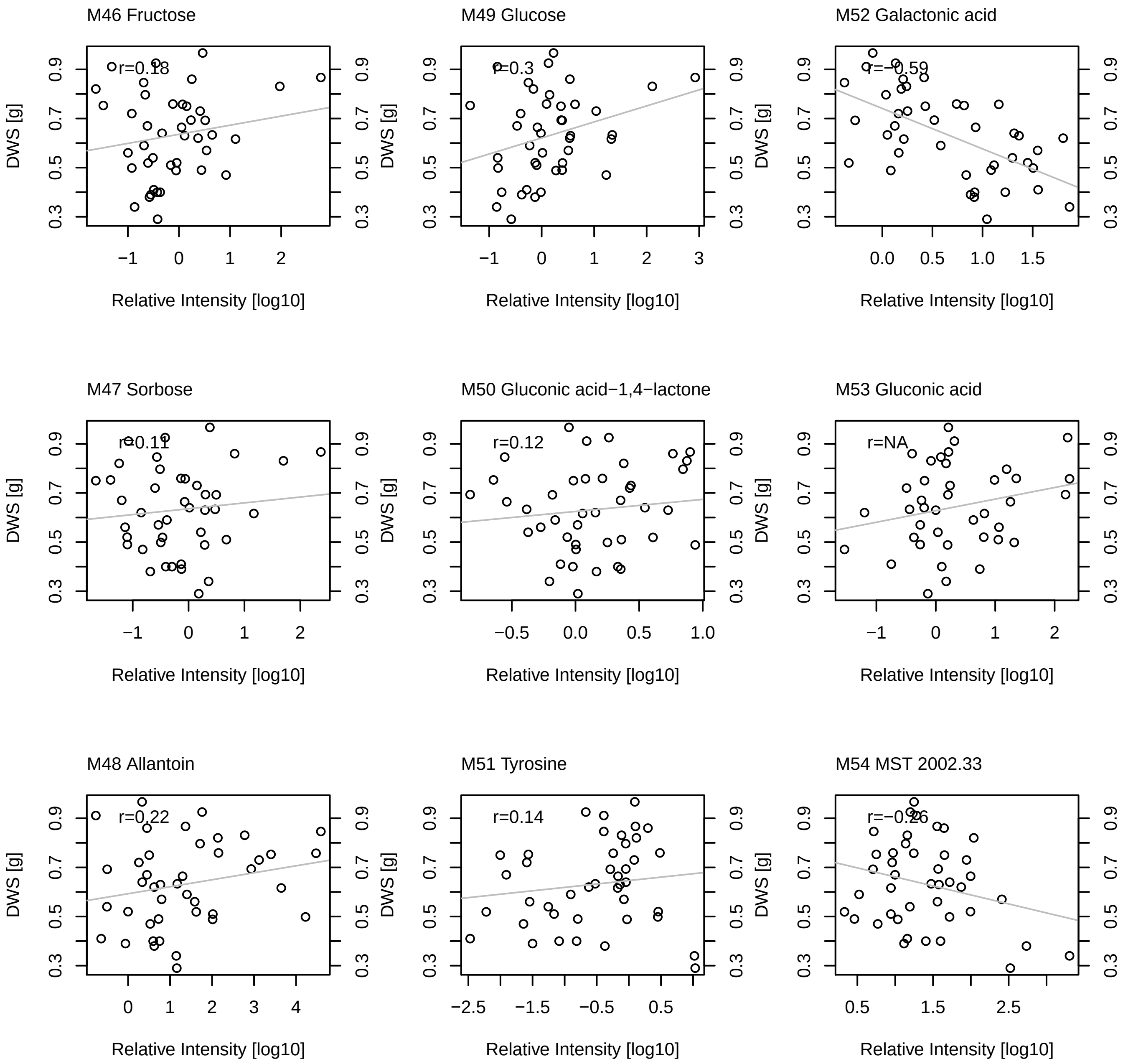
M46 Fructose
r=0.18
M49 Glucose
r=0.3
M52 Galactonic acid
r=−0.59
M47 Sorbose
r=0.11
M50 Gluconic acid−1,4−lactone
r=0.12
M53 Gluconic acid
r=NA
M48 Allantoin
r=0.22
M51 Tyrosine
r=0.14
M54 MST 2002.33
r=−0.26
DWS [g]
Relative Intensity [log10]

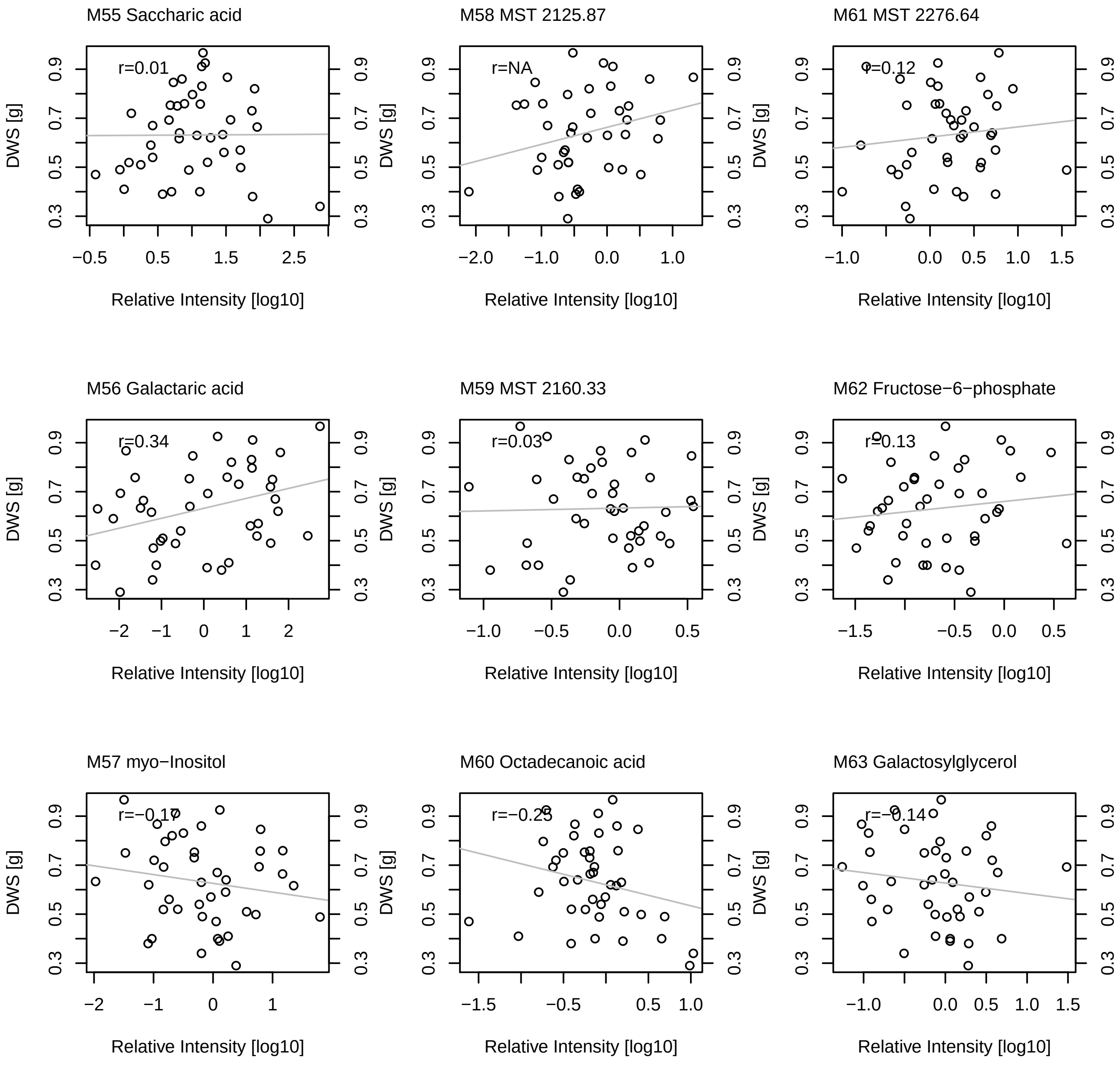

M55 Saccharic acid
r=0.01
M58 MST 2125.87
r=NA
M61 MST 2276.64
r=0.12
M56 Galactaric acid
r=0.34
M59 MST 2160.33
r=0.03
M62 Fructose−6−phosphate
r=0.13
M57 myo−Inositol
r=−0.17
M60 Octadecanoic acid
r=−0.25
M63 Galactosylglycerol
r=−0.14
DWS [g]
Relative Intensity [log10]

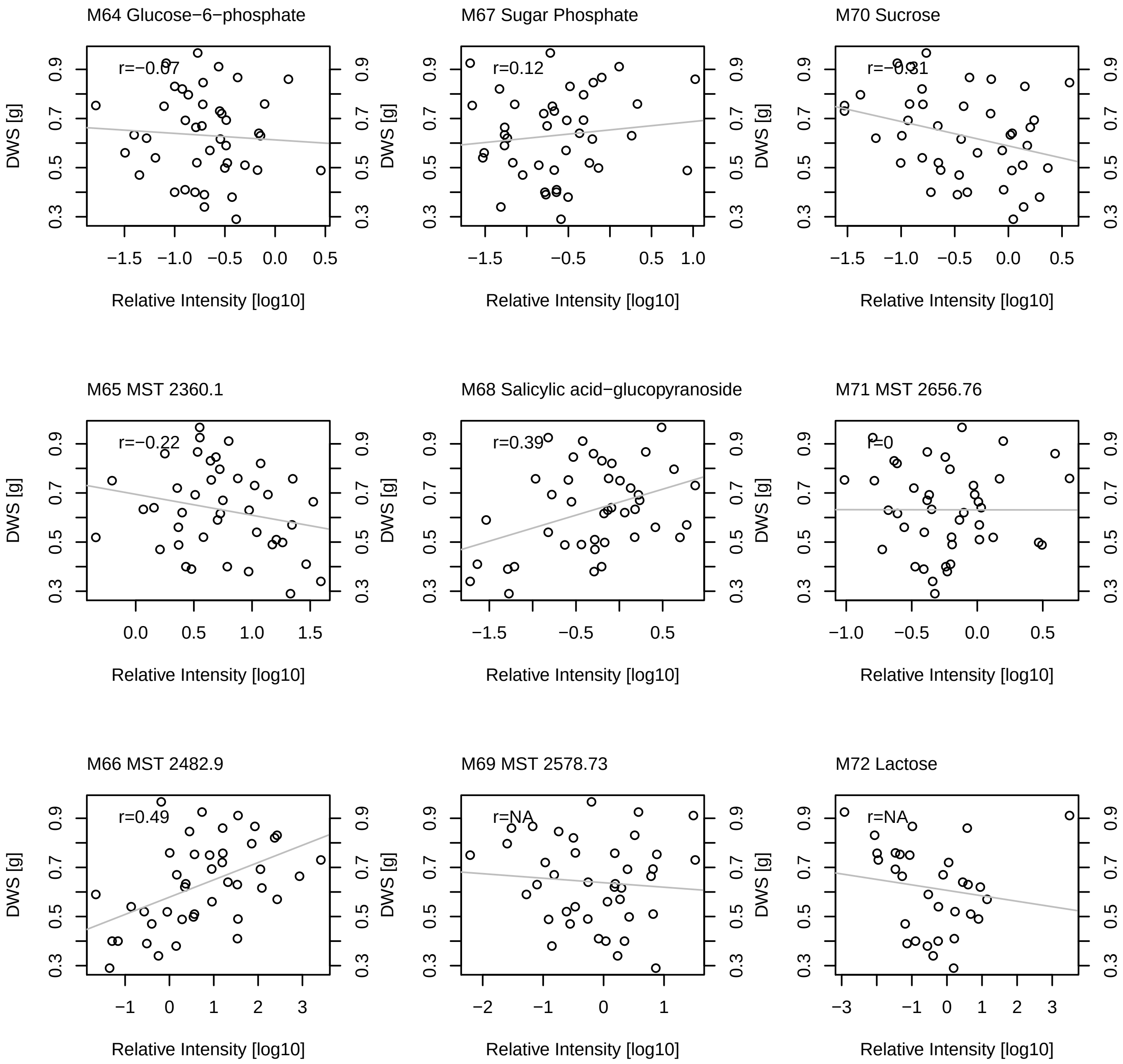
M64 Glucose−6−phosphate
r=−0.07
M67 Sugar Phosphate
r=0.12
M70 Sucrose
r=−0.31
M65 MST 2360.1
r=−0.22
M68 Salicylic acid−glucopyranoside
r=0.39
M71 MST 2656.76
r=0
M66 MST 2482.9
r=0.49
M69 MST 2578.73
r=NA
M72 Lactose
r=NA
DWS [g]
Relative Intensity [log10]

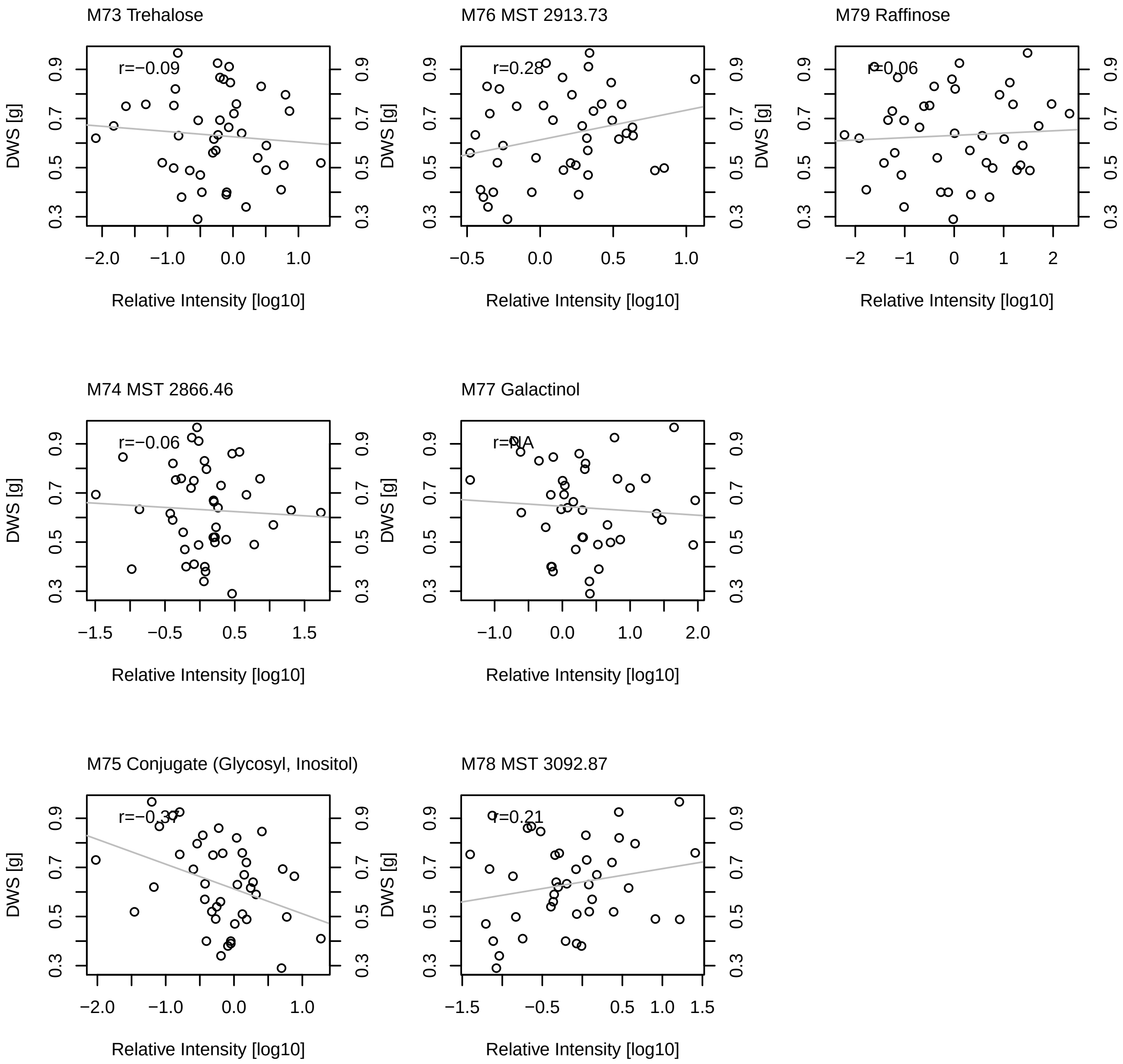
M73 Trehalose
r=−0.09
M76 MST 2913.73
r=0.28
M79 Raffinose
r=0.06
M74 MST 2866.46
r=−0.06
M77 Galactinol
r=NA
M75 Conjugate (Glycosyl, Inositol)
r=−0.37
M78 MST 3092.87
r=0.21
DWS [g]
Relative Intensity [log10]
