# Supplementary material for: Identification of Drought Tolerance Markers in a Diverse Population of Rice Cultivars by Expression and Metabolite Profiling
Source: PLoS One. 2013 May 22;8(5):e63637. doi: 10.1371/journal.pone.0063637 (PMC3661581; doi:10.1371/journal.pone.0063637)
Supplement: Table S3 — Metabolite identifiers (Mid), retention times, metabolite names and loadings of the first five principal components. File Supplemental Table S5.pdf, Format pdf. (PDF) [file pone.0063637.s005.pdf]

Supplemental Table S3

Metabolite identifiers (Mid), retention times, metabolite names and loadings of the first five principal components. File Supplemental Table S3.pdf, Format pdf

MId	Retention time	Metabolite	Loading				
			PC1	PC2	PC3	PC4	PC5
M1	1107,67	Hydroxylamine	-0,061	0,102	0,181	0,245	0,019
M2	1167,53	Monomethylphosphate	-0,155	0,073	0,038	-0,004	0,175
M3	1236,33	Diethyleneglycol	-0,158	0,117	0,088	0,029	-0,106
M4	1262,07	Ethanolamine	-0,109	0,134	-0,004	0,167	-0,081
M5	1264,1	Phosphoric acid	-0,171	0,114	-0,019	0,09	-0,024
M6	1304,47	Glycine	0,16	0,103	-0,024	0,077	0,036
M7	1310,17	Succinic acid	-0,021	0,106	-0,002	0,1	-0,035
M8	1321,67	Glyceric acid	-0,025	0,004	0,168	0,025	-0,083
M9	1335,3	Uracil	-0,103	0,178	0,036	0,029	-0,051
M10	1346,23	Fumaric acid	0,032	0,111	0,148	0,061	-0,009
M11	1353,33	Serine	0,079	0,203	-0,082	-0,008	0,01
M12	1373,43	Threonic acid-1,4-lactone	-0,168	0,081	-0,035	-0,045	0,021
M13	1379,43	Threonine	0,088	0,201	-0,125	0,008	0,029
M14	1419,8	Unknown	0,002	0,163	-0,08	-0,019	-0,125
M15	1426,92	Erythronic Acid 1-4-lactone	-0,035	0,212	0,072	0,052	-0,076
M16	1464,3	Malic acid, 2-methyl-	0,089	0,174	0,061	0,196	-0,011
M17	1477,3	Malic acid	-0,063	0,176	-0,026	0,08	-0,095
M18	1493,2	Erythritol	-0,097	0,042	0,01	-0,264	-0,169
M19	1509,77	Aspartic acid	0,067	0,231	-0,045	-0,048	0,013
M20	1519,43	Pyroglutamic acid	0,175	0,106	0,032	0,061	-0,062
M21	1526	Glutamic acid	0,043	0,069	0,143	-0,121	-0,135
M22	1529,43	Erythronic acid	0,123	0,146	0,043	-0,07	-0,166
M23	1546,43	Threonic acid	0,123	0,161	0,106	-0,096	-0,085
M24	1553,1	Phenylalanine	0,133	0,091	-0,001	-0,121	-0,124
M25	1568,23	Glutaric acid, 2-oxo-	0,083	-0,033	0,091	-0,102	0,054
M26	1574,3	Unknown	0,183	-0,027	0,08	-0,066	0,031
M27	1615,4	Glutamic acid	0,164	0,116	-0,031	-0,024	-0,027
M28	1644,13	Xylose	0,064	0,172	0,058	-0,154	-0,075
M29	1665,8	Asparagine	0,134	0,149	0,009	0,062	-0,092
M30	1687,63	Alcohol (Polyol, Pentahydroxy-)	-0,173	0,037	0,071	-0,122	0,012
M31	1691,2	Unknown	0,069	0,104	-0,122	0,026	-0,142
M32	1699,3	Glucose, 1,6-anhydro, beta-D-	-0,078	0,052	0,214	-0,129	-0,14
M33	1715,23	Ribitol	0,024	0,115	0,077	-0,161	-0,086
M34	1730,77	Unknown	0,162	-0,019	0,112	-0,168	0,065

M35	1732,9	Unknown	0,048	0,099	0,178	-0,192	0,231
M36	1737,17	Putrescine (Agmatine)	0,038	0,097	-0,111	-0,201	0,038
M37	1750,53	Ribonic acid	0,096	0,157	-0,054	-0,062	0,145
M38	1748,53	Glycerol-3-phosphate	0,115	0,108	0,075	-0,037	0,222
M39	1756,27	Unknown	-0,061	-0,053	0,219	-0,124	-0,135
M40	1767,57	Glutamine	0,113	0,15	0,047	0,153	-0,12
M41	1790,17	Glyceric acid-3-phosphate	-0,158	0,097	0,058	-0,024	0,077
M42	1792,47	Shikimic acid	-0,15	0,112	-0,033	0,097	0,121
M43	1804,57	Citric acid	0,06	0,158	-0,023	0,02	-0,05
M44	1816,2	Unknown	-0,125	0,086	0,012	-0,009	-0,017
M45	1843,2	Quinic acid	-0,129	0,117	0,005	0,111	0,137
M46	1856,23	Fructose	-0,082	-0,032	-0,191	-0,225	0,021
M47	1861,54	Sorbose	-0,078	0,099	-0,162	-0,194	0,092
M48	1875,07	Allantoin	0,154	0,047	-0,099	-0,027	-0,034
M49	1881,63	Glucose	-0,025	-0,067	-0,183	-0,259	0,092
M50	1883,57	Gluconic acid-1,4-lactone	0,075	0,026	0,279	-0,039	0,205
M51	1932,6	Tyrosine	-0,088	0,162	-0,013	-0,181	0,044
M52	1984,67	Galactonic acid	0,182	0,056	0,068	-0,018	-0,008
M53	1989,07	Gluconic acid	0,16	0,066	0,03	0,084	0,035
M54	2002,33	Unknown	0,195	0,009	0,037	-0,06	0,02
M55	2002,15	Saccharic acid	0,192	0,011	0,011	-0,048	0,021
M56	2032,47	Galactaric acid	0,021	-0,129	0,237	0,031	0,011
M57	2083,87	Inositol, myo-	-0,049	0,184	-0,211	-0,013	0,14
M58	2125,87	Unknown	-0,111	-0,059	0,006	-0,217	0,029
M59	2160,33	Unknown	-0,047	0,033	-0,269	-0,079	0,044
M60	2240,67	Octadecanoic acid	-0,068	0,161	0,131	-0,093	-0,195
M61	2276,64	Unknown	0,1	-0,002	0,032	-0,025	0,193
M62	2292,77	Fructose-6-phosphate	-0,171	0,084	0,075	-0,07	0,092
M63	2297,2	Galactosylglycerol	-0,042	0,034	0,206	0,053	-0,022
M64	2307,1	Glucose-6-phosphate	-0,181	0,103	0,038	-0,042	0,081
M65	2360,1	Unknown	0,181	0,056	-0,026	-0,036	0,065
M66	2482,9	Unknown	0,145	-0,011	-0,014	-0,128	0,143
M67	2552,47	Phosphate (Sugar)	-0,17	0,064	0,085	-0,068	0,079
M68	2565,97	Salicylic acid-glucopyranoside	-0,061	-0,04	0,284	-0,049	0,051
M69	2578,73	Unknown	-0,036	0,111	-0,089	-0,087	-0,099
M70	2629,63	Sucrose	-0,135	0,157	0,006	0,074	-0,069
M71	2656,76	Unknown	-0,107	0,147	0,055	-0,09	0,077
M72	2678,6	Lactose	-0,087	0,01	0,157	-0,118	-0,045
M73	2730,07	Trehalose, alpha,alpha-	-0,077	0,127	-0,043	-0,057	0,12
M74	2866,46	Unknown	0,078	0,027	0,109	-0,196	0,032
M75	2907,17	Conjugate (Glycosyl, Inositol)	-0,029	0,159	-0,145	0,075	0,162
M76	2913,73	Unknown	0,072	0,081	0,055	-0,141	0,103

M77	2972,97	Galactinol	0,084	0,08	0,079	0,116	0,302
M78	3092,87	Unknown	-0,072	-0,016	0,13	0,024	0,262
M79	3362,87	Raffinose	0,016	0,087	0,037	0,147	0,3
