# Supplementary material for: Identification of Drought Tolerance Markers in a Diverse Population of Rice Cultivars by Expression and Metabolite Profiling
Source: PLoS One. 2013 May 22;8(5):e63637. doi: 10.1371/journal.pone.0063637 (PMC3661581; doi:10.1371/journal.pone.0063637)
Supplement: Table S4 — List of qPCR primer sequences that were used for quality checks and RT-PCR together with their Primer Identifier (PId), TIGR Locus identifier (Locus ID), OligoID from the gene chip (see Degenkolbe et al. 2009), and direction (FW = forward, RV = reverse). File Supplemental Table S6.pdf, Format pdf. (PDF) [file pone.0063637.s006.pdf]

Supplemental Table S4

List of qPCR primer sequences that were used for quality checks and RT-PCR together with their Primer Identifier (PId), TIGR Locus identifier (Locus ID), OligoID from the gene chip (see Degenkolbe *et al.* 2009), and direction (FW = forward, RV = reverse).

PrimerID	Locus ID	OligoID	Direction	Sequence
	Os03g50890		FW	CTCCCCCATGCTATCCTTCG
			RV	TGAATGAGTAACCACGCTCCG
	Os03g50890		FW	TGGAGGATCCATCTTGGCAT
			RV	TCGTACTCAGCCTTGGCAATC
	Os03g50890		FW	TACCCCATCGAGCATGGTATC
			RV	TTCTCACGATTGGCCTTGG
	Os08g19610		FW	CCACCATCACAGATCGGATCTT
			RV	GCGGTCAGAGCGAAAGTAGCTA
P1	Os01g04860	TR000232	FW	CTCAATCCGATTGGAACAGGC
			RV	AGCCAATGCGCTTGCCATA
P2	Os01g06310	TR000321	FW	GCGGAAAGTGGAAGTGAAGC
			RV	CGTACGCCACATGGTTGATACT
P3	Os01g06740	TR000345	FW	GATCCTGCTGTTCGTCGAGAAG
			RV	GCTGTTCGATCAAGCAAGCTG
P5	Os01g60260	TR002928	FW	TCAACCTATGCCTGTGCAACA
			RV	CCGAAACGCAAAACCAGAAG
P6	Os01g63060	TR003102	FW	ACGTGTGCAGATGAACGGAA
			RV	ACTAGACAAGCCGCCTGATGA
P8	Os01g64660	TR003185	FW	CAAGATTCACGAGAGATCCCCA
			RV	ATTCGCACGCCTTGATGGT
P9	Os01g72370	TR003619	FW	CACCAACTGCAAACCAGGAGT
			RV	CCGCCACATTGCTCAACAA
P10	Os02g41470	TR005088	FW	TAAGCGTGCTGCAATGACCA
			RV	AAGCAGGTGGCATTCCGTACT
P11	Os03g03510	TR006178	FW	TGCTGCATTCGATTGTTCAGAG

			RV	GGTTGAATCCCTGTGACCTTGA
P12	Os03g04410	TR006243	FW	ATCACATCTCACCTGCCGGAA
			RV	TCGCCGACTGCCATATGAGTT
P13	Os03g04710	TR006269	FW	TGGTAGTCTCGCGTTCATTTCA
			RV	CGTCGTAAACTTGATTCCGCTT
P14	Os03g11900	TR006722	FW	TGTGGAAGCAGCACTGGTTCT
			RV	CGACTAAATTAAACGGTGGCG
P15	Os03g14990	TR006919	FW	GAGGTGATTCGATTGGTGGTG
			RV	TTTCGCCAATTCAGCCTCA
P16	Os03g16050	TR006994	FW	CCAAAATCTTGACCAGGTGGA
			RV	ATGGTGAGCACGAAGATGACC
P17	Os03g18130	TR007134	FW	TCGCTGACCACCTAGGAACCAT
			RV	ACAGCACCATCTTAACCCCGAG
P18	Os03g20100	TR007277	FW	TGCAACCAGGAGATACGCTCA
			RV	TGGATTGCGAATCATGTCACC
P19	Os03g21370	TR007351	FW	TTTTGGAAGATTGTGGACGGC
			RV	GAAGACTGCATTTTTCCCGTGA
P20	Os03g22620	TR007427	FW	GCCGGTGATTTAGCAACAAAAG
			RV	GAATGCAGCCAAGTCATCCTG
P21	Os03g37490	TR007848	FW	AGTCGGATTCAACGCAGCA
			RV	AAAGAACGACAGCACCGTCAC
P22	Os03g40020	TR007941	FW	TCAAGATCTGTGCACGCAGTG
			RV	CAAGAGGCAGTTGGTGGAAAA
P23	Os03g44810	TR008098	FW	TGTCTTTGCTCATGCTGTGCAT
			RV	ATCTCATTCCGCTCTTCTCGC
P24	Os03g56930	TR008765	FW	GTGCAGTATGTGTTTGTGGCG
			RV	GCATGCGTGGTAATTTGCC
P25	Os03g57640	TR008795	FW	GCTTTCTGTGGCATTGCGT
			RV	CCAATGTCCAATAGGCTTCCA
P26	Os03g58400	TR008838	FW	TGCCCAACACAGCCAAAAG
			RV	TTCCCCATTGGCTGAGGAT
P27	Os03g60100	TR008946	FW	GTTGTTCGCTGAGGTTGCAGA
			RV	AAGCCATAGGTGCATTGTCTCC

P28	Os03g62630	TR009108	FW	AATGAGGCTCAATGCTGACGAC
			RV	CCCCAAAACATGGAAACCTGA
P29	Os04g38680	TR010306	FW	TGTCAACTACGGATCCATGGC
			RV	TTCGAGCTGATCTTTCCCTCC
P30	Os04g52090	TR011061	FW	CCCCACAAATCCCTCGTAGAA
			RV	CCGTGCAAAAAGGACACGAT
P31	Os04g53210	TR011145	FW	GTTGCAGTGATCGAATTGTCCA
			RV	CCTTATTACCCGGCATCATCAA
P32	Os04g55600	TR011237	FW	ATGCGTCCTCTCCGATTGGTT
			RV	TAGGAGCCGCAGCTGACAAAT
P34	Os04g55710	TR011248	FW	GCAAAGCCTAGGAGCACTGAAA
			RV	ACAGTGCCACGGAGTAGTTGGT
P35	Os04g57550	TR011361	FW	TGCATCATTACAGTACCGCTCG
			RV	TCTCGATACCGACACCAAGGTC
P37	Os05g39250	TR012002	FW	TCGGCAACAAGGTGACCAA
			RV	AAAATGCAGAGAGCTCGCGTC
P38	Os05g46480	TR012195	FW	CGCCGTGAATGATTTCCCT
			RV	CCACCACTTCATACAGCATCGA
P39	Os06g08720	TR012608	FW	CTTCAATCTGGCTGAGGTGCA
			RV	TGCAGCACAGATCTTTCGCA
P40	Os07g02330	TR012706	FW	CTACAGCTTGTACAACTCCAC
			RV	GTGTTAACCTAACTCGACATCC
P41	Os07g02710	TR012727	FW	TCTGAGACGGTCTGACGGATTC
			RV	AGATATAGGCAAGCGGCAGGA
P42	Os07g04930	TR012858	FW	CATAATTAAGGACGCTGCCA
			RV	AAATACTTATAGCCGCGCAC
P43	Os07g08840	TR013075	FW	TCGCAGGCGCAAGTTATCA
			RV	TGATATTCGAGGACACCCAAGC
P44	Os07g15460	TR013365	FW	ACATTCGCCAACGTGCTCAT
			RV	GCTCACGACGAGACACGAACTT
P46	Os07g44410	TR014542	FW	GCGACAGATGATGATCGATGA
			RV	CGTACTAGCCCACACAATCAGG
P47	Os07g47590	TR014718	FW	CAGTTGCTCTTTCCTGTGGTCA

			RV	CACTTTGATTTGCGCTCGG
P48	Os07g47990	TR014740	FW	TCGTCTGCTCCAAGGTGAATT
			RV	CCAAGAAAGCGTGAACATGATC
P49	Os07g49270	TR014832	FW	GCCCAACAAAACACATGCCTAC
			RV	GGACTCACGAAGCTTGTTCAGC
P50	Os08g02490	TR014937	FW	GGCATCTCCTGCTCAGTCGATA
			RV	GCAGCAGTGACAGACCAGGAAT
P53	Os12g37690	TR020013	FW	ATGCCAGTGATGACATCGTCG
			RV	CCCACCATTCACCATCTCATTG
